# Diel expression dynamics in filamentous cyanobacteria

**DOI:** 10.1128/mbio.03779-24

**Published:** 2025-11-18

**Authors:** Sarah J. Kennedy, Douglas D. Risser, Blair G. Paul

**Affiliations:** 1 Bay Paul Center, Marine Biological Laboratory, Woods Hole, Massachusetts, USA; 2 Department of Biology, University of Colorado Colorado Springs, Colorado Springs, Colorado, USA; Johns Hopkins University Bloomberg School of Public Health, Baltimore, Maryland, USA

**Keywords:** cyanobacteria, diel cycle, regulation of gene expression, hypermutation, retroelements, prophage

## Abstract

Filamentous cyanobacteria of the *Nostocaceae* family can differentiate into multicellular forms to adapt to environmental stresses, and members can establish symbiosis with various embryophytes. Representative laboratory strains are typically grown under continuous light to maintain stable metabolic conditions; however, this departure from a natural diel cycle can result in extended stress. Early genomic examination of *Nostoc punctiforme* suggests the genetic potential for a circadian clock, but we lack insight into global cellular dynamics through the natural diel cycle for this model organism. Here, we comprehensively assess changes in expression of core cellular processes and the mobilome of accessory genetic elements during diel growth of *N. punctiforme* PCC 73102. The primary transcriptome confirmed that multicellular cyanobacteria precisely coordinate photosynthesis and carbon assimilation for cell division during the day, while control of DNA recombination and repair appeared to be sequestered to darkness. Moreover, we expanded the known repertoire of light-sensing proteins to uncover a putative regulator of circadian rhythm that itself exhibits striking oscillation between day-night expression. This was in sharp contrast to the arrhythmic pattern observed for a homolog of the canonical circadian input kinase in unicellular cyanobacteria. Looking beyond cellular coordination of diel growth, we uncovered dynamic mobile elements and, notably, targeted hypermutation by retroelements that are likely maintained for conflict mitigation, which is crucial for a multicellular lifestyle.

**IMPORTANCE:**

Model strains of filamentous cyanobacteria are typically cultivated under controlled laboratory conditions that poorly reflect the natural environment, including growth under constant light. Our study addresses this discrepancy to provide a new benchmark for investigating gene expression in the model organism, *Nostoc punctiforme*. By analyzing changes in the global transcriptome over a diel cycle, we found a clear partition of cellular processes between periods of light and darkness, with metabolism dominating in the light and cell maintenance and repair processes dominating in the dark. In addition, an active mobilome of genetic elements was uncovered with dynamic expression patterns throughout a diel cycle. Our findings highlight the importance of considering diel cycles in cyanobacterial research and provide new insight into the regulatory complexity, genome plasticity, and adaptive mechanisms of these ecologically important organisms. Our study reinforces the need to consider the natural diel cycle in laboratory models of filamentous cyanobacteria, bringing new insights into their regulatory complexity and revealing adaptive drivers of genome plasticity that may enable members of *Nostoc* to occupy a wide variety of ecosystems.

## INTRODUCTION

Multicellular cyanobacteria, particularly those from taxonomic subsections IV and V, are thought to have evolved in several independent stages of life’s history and occupy a vast range of environments (1, 2), where they adapt to variable stresses and serve as key drivers of ecosystem resilience. These microorganisms can differentiate several distinct cell types, including nitrogen-fixing heterocysts that shield the inside of the cell from oxygen (3), motile hormogonia that are thought to establish symbiosis with plant hosts (4), and desiccation-tolerant akinetes that develop and undergo dormancy in response to nutrient limitation or other stresses (5). *Nostoc punctiforme* strain PCC 73102 (ATCC 29133) is a model organism for studying the development of these specialized cell types (6–9).

Filamentous and multicellular cyanobacteria have been investigated, in large part, through experimental manipulation of model strains that represent members of the family *Nostocaceae* (4, 10). Growth experiments with *N. punctiforme* PCC 73102, *Nostoc sp*. strain PCC 7120, and other model representatives are typically carried out under continuous light (i.e., 24-h constant illumination), as opposed to mimicking the natural light-dark diel cycle. Constant light incubations have been used in the past for examining the ability of various cyanobacteria to fix nitrogen (11), maintain photosystem regulation (12), establish circadian rhythm (13, 14), and undergo chromosome supercoiling (15). Yet, continuous light exposure has been linked to oxidative stress and upregulation of protective response pathways (12). The departure from the natural diel cycle to continuous light induces global transcriptional changes in cyanobacteria, complicating the interpretation of gene expression due to stress-related perturbations and adaptive responses. Moreover, transcriptomic studies with *N. punctiforme* have not yet addressed the natural cellular response to a diel cycle and, in turn, whether the organisms can establish circadian rhythm from long-maintained cultures. We thus lack a complete view of global regulation during natural growth of model filamentous cyanobacteria.

While *N. punctiforme* PCC 73102 has been studied as a model for understanding the molecular basis of multicellularity and symbiosis in filamentous cyanobacteria, *Synechococcus elongatus* PCC 7942 has been the primary model organism to study bacterial circadian rhythm. As such, most details for the circadian clock and genetic pathways that are regulated on a diel cycle are based on this unicellular cyanobacterium. The core clock components, including KaiA, KaiB, and KaiC proteins, and the input/output atypical kinase, CikA, are each found in the genome of *Nostoc punctiforme* and in other members of the *Nostoc* genus, but these orthologs differ in sequence and domain composition (16, 17). Recently, Arbel-Goren and colleagues found that the ability to fix nitrogen was lost in mutant strains of filamentous cyanobacteria that lacked the core clock genes, while also demonstrating that differentiation of heterocysts occurred with specific timing on a circadian cycle (18). While circadian control of gene expression may differ between unicellular and multicellular cyanobacteria, a comprehensive transcriptomic analysis during a natural circadian cycle still has not been carried out for any members of filamentous cyanobacteria. *N. punctiforme* may possess genes for either clock synchronization or output control that are distinct from homologs of known circadian input-output proteins of *S. elongatus*. Expression of such genes may be tied to either (i) a diel shift in photosynthesis (i.e., the redox state of quinones), providing input to the clock, or (ii) oscillation that results from clock output and control by the master regulator RpaA.

The genomes of multicellular cyanobacteria are notably expansive, especially when compared with their unicellular counterparts that maintain smaller genomes (19, 20), and this trait has been attributed to rampant duplication and horizontal gene exchange. Notably, the functional roles and mechanisms that underlie the expansion of diverse mobile elements and variable repertoires of protein kinases in multicellular cyanobacteria remain to be characterized. The balance between maintenance and differential expression for multiple gene copies raises questions on the evolutionary fate of these expanding loci (21).

In this study, we established diel entrainment of *N. punctiforme* and generated a global gene expression data set, from which we systematically uncovered dynamic cellular processes that oscillate through the light-dark cycle. This study provides a new framework and primary resource for interpreting dynamic gene expression in filamentous cyanobacteria undergoing diel responses to light while establishing circadian rhythm.

## RESULTS AND DISCUSSION

**Fig 1:**
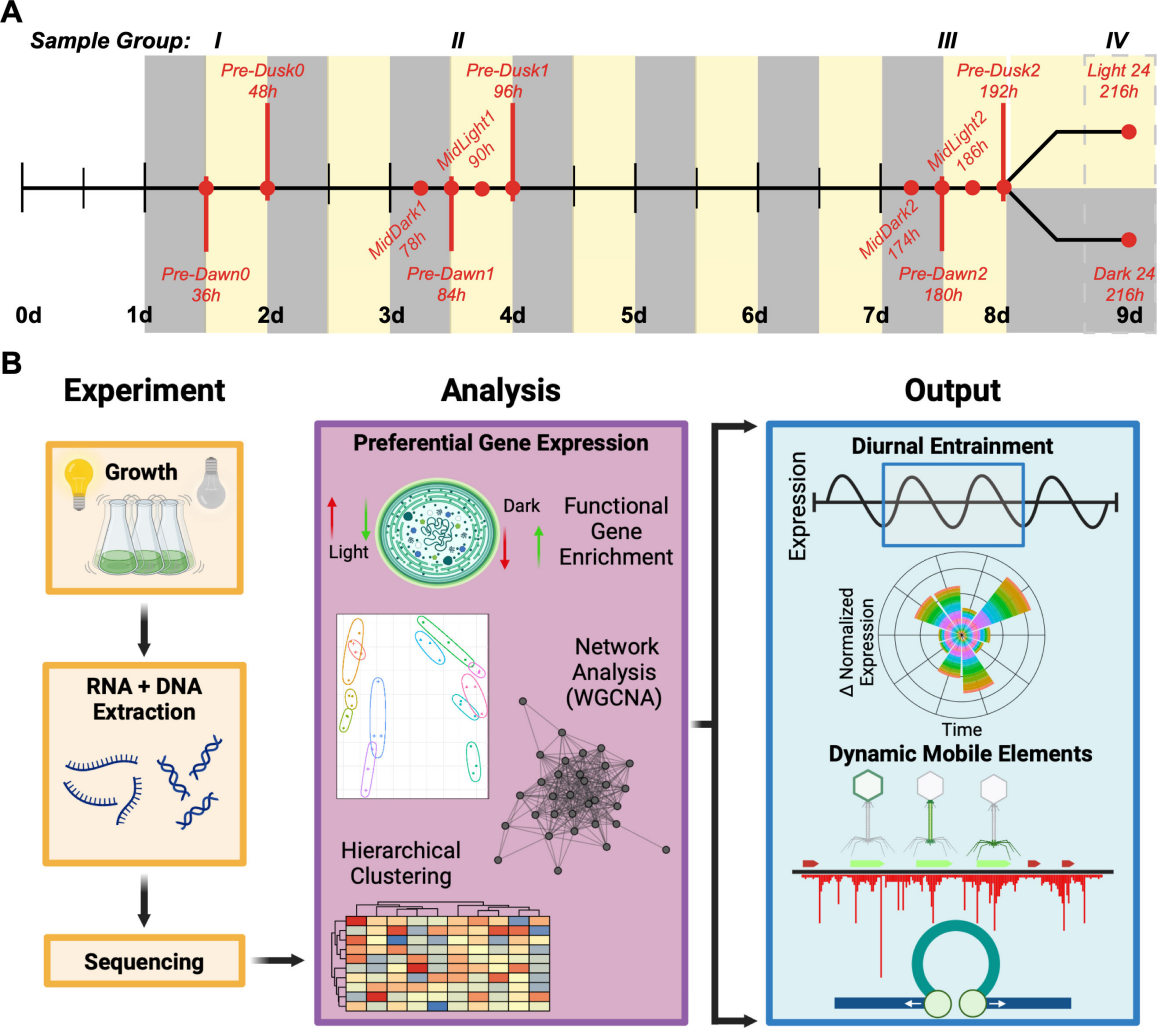
Overview of experimental design and analytical workflow. (**A**) Schematic of the experimental design and timeline. Sampling spanned 9 days, including 12 time points to capture pre-dawn, pre-dusk, mid-dark, and mid-light phases. Four sampling stages are indicated (I–IV) that encompassed early diel entrainment, late diel entrainment, and reversion to continuous light or dark. Individual time points are highlighted with the respective light or dark state (periods of dark are indicated by shaded boxes). (**B**) workflow illustrating the experimental design and analysis. Experimental steps include diel growth, RNA and DNA extraction, and sequencing. The preferential gene expression workflow included functional gene enrichment, statistical analyzes, weighted gene co-expression network analysis (WGCNA), and hierarchical clustering. The workflow outputs revealed gene expression dynamics during diel entrainment and active mobile elements, such as phage and transposons. Images created with BioRender.

To uncover dynamic cell processes during diel growth in *N. punctiforme*, we carried out a large-scale growth and multi-omics experiment, comprising genome re-sequencing and 36 transcriptomic libraries. Cultures were grown on a light-dark (LD) diel cycle (i.e. 12 h light followed by 12 h dark), and four distinct stages were targeted to generate transcriptomes (Fig. 1A). In the first stage, two time points were harvested after 36 and 48 h—referred to here as “Pre-Dawn0” and “Pre-Dusk0,” respectively. Next, the second and third stages were intended to observe early versus late diel entrainment, separated by three days. To this end, both the early and late entrainment stages were sampled at 6-h intervals encompassing: (i) mid-phase dark, (ii) 30 min pre-dawn, (iii) mid-phase light, and (iv) 30-min pre-dusk. In the fourth stage, diel cultures grown for 8 days were incubated for a final 24 h in either (i) continuous light (LL) or (ii) continuous dark (DD). Global RNA expression was assessed for all four sampling stages, comprising 12 time points measured in triplicate (Fig. 1B).

From a global perspective, the individual transcriptomes (*n* = 36) are most similar among replicates (Fig. S1), then primarily separate according to growth in the light vs dark (Fig. S2). Broadly, transcriptomes from the light phases (Pre-Dusk, MidLight, Light24) contrast with those from dark phases (Pre-Dawn, MidDark, Dark24), while within-phase clusters of individual samples differentiate among the respective time points. Moreover, multiple distinct temporal clusters emerge from this global analysis: (i) initial dark, (ii) initial light, (iii) mid-light, (iv) pre-dusk, (v) mid-dark, (vi) pre-dawn, (vii) 24-h post in dark, and (viii) 24-h post in light.

### Global response to light availability

**Fig 2:**
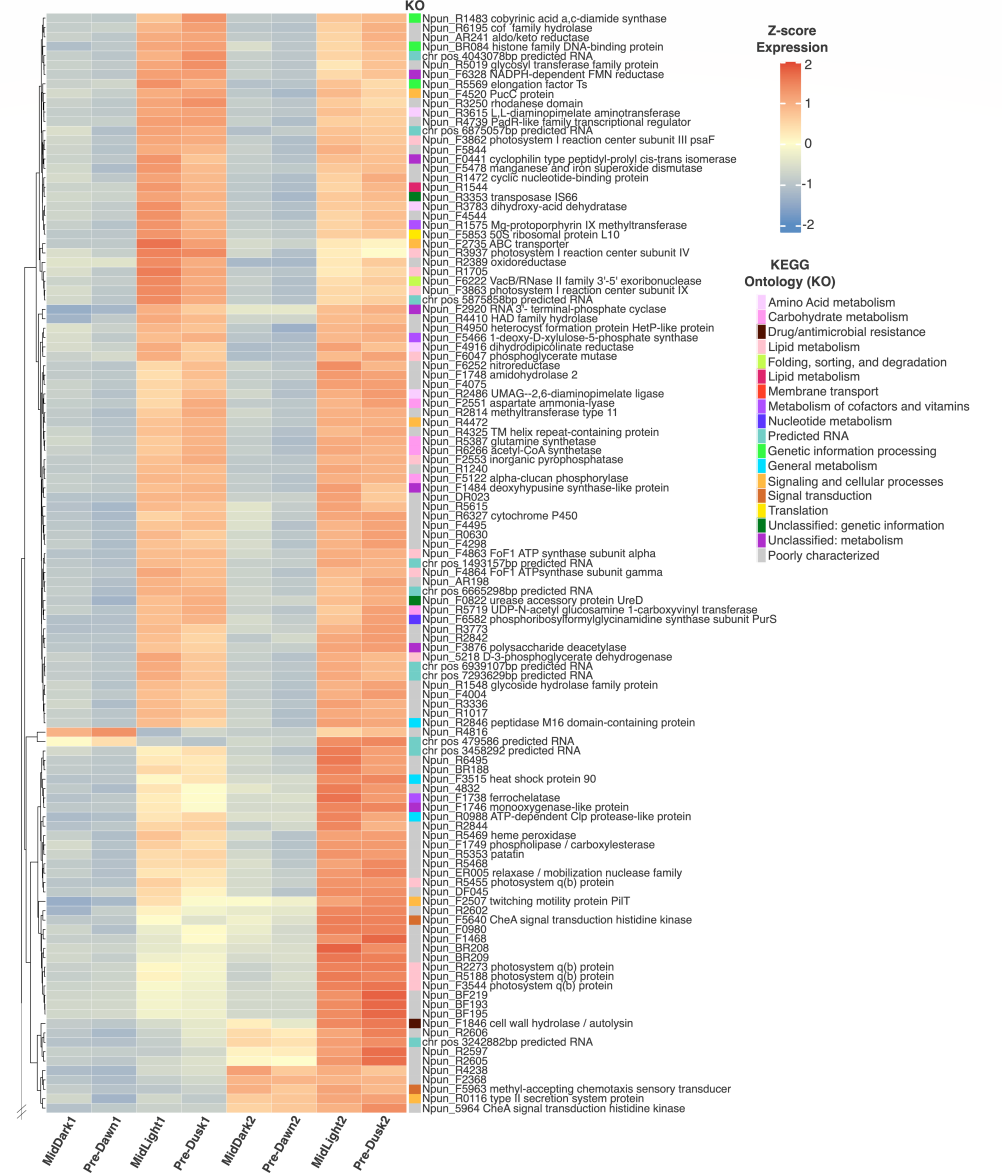
Preferential gene expression during the subjective day across diel time points. Heatmap of gene expression from sampling stages II and III (MidDark1, Pre-Dawn1, MidLight1, Pre-Dusk1, MidDark2, Pre-Dawn2, MidLight2, and Pre-Dusk2). The data were hierarchically clustered using Euclidean distance and complete linkage. Each tip on the dendrogram represents a gene, with z-score normalized expression indicated as either blue (i.e., downregulation) or red (upregulation). KEGG annotations are represented with distinct colors corresponding to their functional categories.

**Fig 3:**
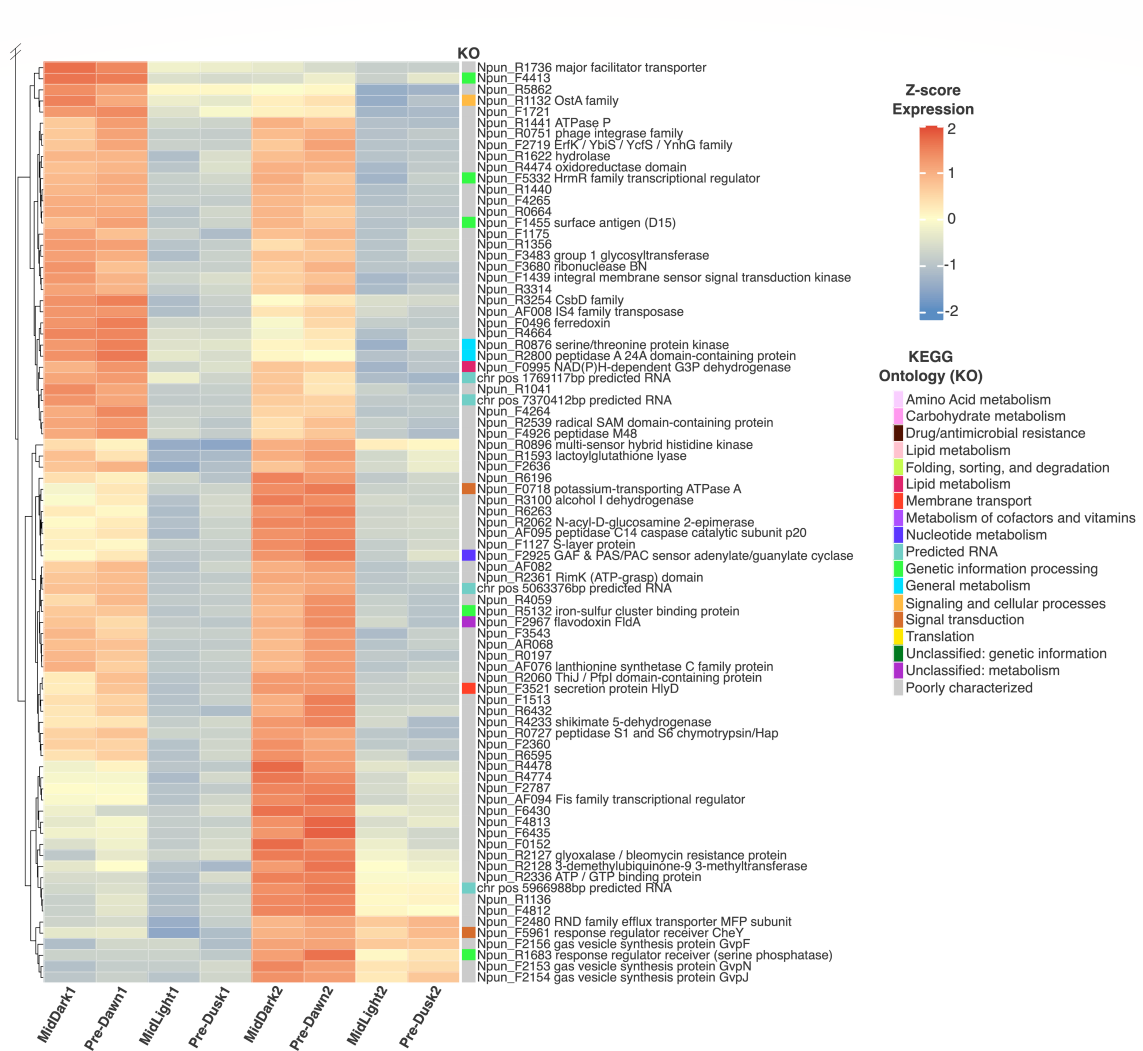
Preferential gene expression during the subjective night across diel time points. The heatmap displays gene expression from sampling stages II and III (MidDark1, Pre-Dawn1, MidLight1, Pre-Dusk1, MidDark2, Pre-Dawn2, MidLight2, and Pre-Dusk2). The data were hierarchically clustered and displayed using the same approach as Fig. 2. For z-score expression values, blue indicates downregulation, and red indicates upregulation. KEGG annotations are represented with distinct colors corresponding to their functional categories.

To assess diel gene expression in *N. punctiforme*, we first looked at the core periods of our time series, which encompassed two full 24-h LD cycles (i.e., stages II and III; Fig. 1A). Hierarchical clustering of transcriptomes for these core time points revealed two overarching partitions, apparently corresponding to the light/dark state. By assessing expression variation across groups of “Light” (Mid-Light and Pre-Dusk) and “Dark” (Mid-Dark and Pre-Dawn) time points, we found 200 genes and predicted RNAs that had significantly different expression profiles across the core diel entrainment. Among this subset, 117 genes/RNAs were preferentially expressed within mid-light and pre-dusk time points (Fig. 2). This set of genes was associated with diverse metabolic pathways; broadly, these include general energy, carbohydrate, amino acid, and other core pathways—including metabolisms of lipids, nucleotides, and cofactors/vitamins. Notably, preferential gene expression was most similar between adjacent time points (e.g., MidLight1 and Pre-Dusk1), rather than by condition (e.g., MidLight1 and MidLight2; Pre-Dusk1 and Pre-Dusk2). In contrast, 83 genes and predicted RNAs were preferentially expressed within dark-associated conditions, with the majority of gene functions remaining poorly characterized (Fig. 3).

Although *N. punctiforme* forms nitrogen-fixing heterocysts, our diel experiment was not designed to assess differential gene expression under nitrogen-fixing conditions, as has been carried out in previous transcriptomic studies (22). Nonetheless, we examined the diel transcriptome for genes involved in nitrogen fixation, initiation of heterocyst differentiation, commitment, maturation, and envelope glycolipid formation (23). The nitrogenase (nif) cluster genes and others putatively involved in N-fixation were not correlated with either the light or dark time points. One gene (Npun_R4950) that encodes a HetP-like protein showed preferential expression during the subjective day, with peak expression in MidLight and PreDusk time points (Fig. 2), but we could not find a clear association with light or dark stages for other genes involved in heterocyst development. This reflects asynchronous heterocyst development during the diel time series, given the lack of a nitrogen step-down in the cultures.

Looking beyond the core LD time points, we next clustered the full experimental transcriptional data set, which revealed “light-'” and “dark-”associated expression (Fig. S3). Specifically, we carried out weighted gene co-expression network analysis (WGCNA) for all 12 time points, resulting in gene modules that display co-regulation and thus may underlie common cellular functions (Fig. S4A). Importantly, the WGCNA modules were analyzed and grouped based on their correlations with time points (Fig. S4B), which were used to determine preferential expression. Modules that were associated with light comprised 1,035 genes, whereas the dark-associated modules comprised 776 genes. Most of the light-associated genes were assigned to Light24 modules (507 genes), followed by Pre-Dusk (326 genes) and MidLight (202 genes) modules. Conversely, the majority of dark-associated co-expression was identified within Pre-Dawn (483 genes), followed by Dark24 (164 genes) and MidDark (129 genes) modules.

### Light-associated co-expression of metabolic pathways and motility systems

**Fig 4:**
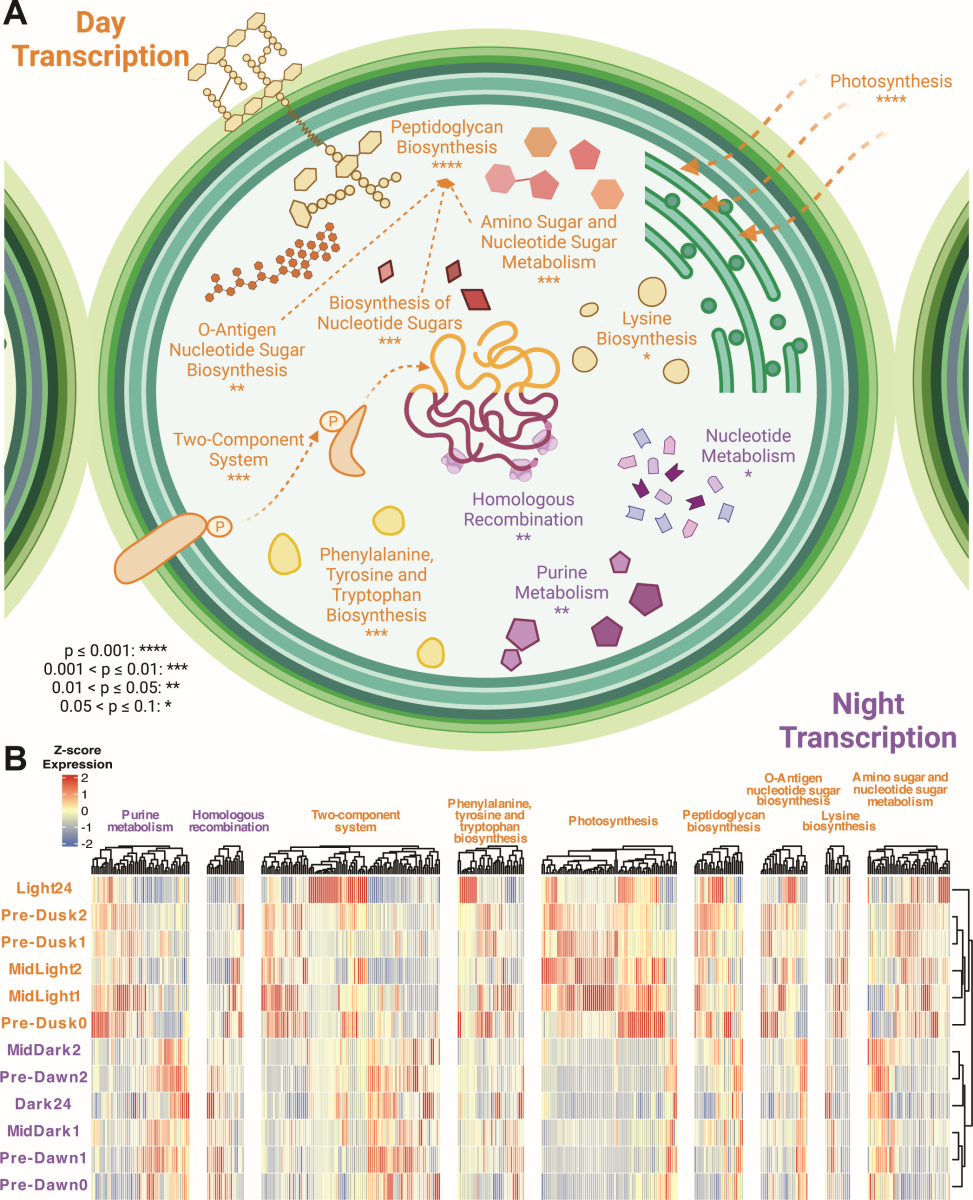
Gene expression and pathway enrichment analyses of diel transcription. (**A**) Cell process diagram depicting enriched pathways associated with day (orange) and night (purple) transcription. Significance levels (hypergeometric test) are indicated as follows: ****P ≤ 0.001, ***0.001 < *P* ≤ 0.01, **0.01 < *P* ≤ 0.05, *0.05 < *P* ≤ 0.1. (**B**) Heatmap of hierarchically clustered gene expression profiles (Euclidean distance, complete linkage) within enriched pathways from the time-course experiment. Z-score normalized expression levels are color-coded, with red indicating upregulation and blue indicating downregulation.

Having conducted separate analyses of global expression clustering (hierarchical), variation (ANOVA), and correlation (WGCNA), we next used the combined output of associated genes as input for functional enrichment analysis (Fig. S3). Importantly, ANOVA was able to identify genes with significantly dissimilar expression between light and dark time points, including those genes not recognized as part of a WGCNA module (WGCNA is more sensitive to specific time points). Next, we carried out gene set enrichment analysis (GSEA) with all these genes uncoupled and reanalyzed by their normalized expression across time points, thus remaining agnostic to whether either ANOVA or WGCNA grouped them. GSEA revealed light-associated convergence of biosynthetic pathways, including synthesis of peptidoglycans, O-antigen nucleotide sugars, amino sugars, and other nucleotide sugars (Fig. 4). Unsurprisingly, photosynthesis was the most significantly enriched pathway within light-available transcriptomes (particularly within Pre-Dusk and Mid-Light, Fig. 4A). Peptidoglycan biosynthesis was also enriched within Pre-Dusk and Mid-Light transcriptomes. While several peptidoglycan biosynthetic enzymes are shared among other light-enriched sugar metabolic pathways (Fig. S5), this apparent co-expression (Fig. 4B; Fig. S6) suggests concerted regulation when *N. punctiforme* is entrained in a diel cycle.

Insights from prior studies on diel growth in *Synechococcus elongatus* point to the importance of glycogen cycling (24) for continued LD cycle survival (25). Closer examination of the diel transcriptome reveals the specific enzymatic pathways linking metabolism with cell wall biosynthesis. Notably, our time series revealed enrichment of enzymatic pathways intertwining glycolysis/gluconeogenesis intermediates (specifically, glucose-6-phosphate [Glc-6P] and fructose-6-phosphate [Fru-6P]) with other sugar metabolic pathways, which appear to provide the necessary precursors for peptidoglycan biosynthesis (Fig. S5).

While these specific enzymes are shared among pathways that apparently respond to a diel cycle (O-antigen nucleotide sugars; amino sugars; and nucleotide sugars), expression of two genes (*nagB,* Npun_R1302; *glmU,* Npun_F0907) correlated with multiple key peptidoglycan biosynthetic genes (Table S1). The gene *nagB*, which catalyzes conversion of glucosamine 6-phosphate (GlcN-6P) to Fru-6P, showed apparent co-expression with both a low molecular weight PBP (LMW PBP, Npun_R1733, ~ 0.87) and an isoprenyl transferase (*uppS,* Npun_R1952, ~ 0.76). Conversely, *glmU*, which is responsible for catalysis of the last two steps of *de novo* biosynthesis of peptidoglycan precursor UDP-N-acetylglucosamine (UDP-GlcNAc), correlated with five peptidoglycan biosynthetic enzymes. These include the genes responsible for the first committed step of peptidoglycan biosynthesis (*murA*, Npun_R5719, ~0.86), precursor transport/insertion into the cell wall (*bacA*, Npun_R4507, ~0.83), and crosslinking (LMW PBP, Npun_R2557, ~0.83; Class B PBP, Npun_F4453, ~0.79; Class B PBP, Npun_F0907, ~0.77).

Critical cell division genes (*ftsE, minC, ftsK, cdv1,* and *minD*) were highly correlated (>0.90) with several direct peptidoglycan synthetic genes (class B PBP, *murE,* LMW PBP) and indirectly synthetic/convergent metabolic genes (*ddl*, *glmU*; Table S2). Together, these genes tied to cell division appeared correlated in terms of their expression across the diel cycle. Tight co-expression of sugar metabolism, peptidoglycan biosynthesis, and cell division suggests that cell wall growth and development follow precise timing mechanisms linked to the LD cycle, as previously demonstrated by circadian clock-gated cell division in *S. elongatus* (26).

Metabolomic studies in *S. elongatus* entrained in diel growth are consistent with our transcriptional observations*,* wherein daytime accumulation of various sugar metabolites is coordinated with increased rates of cellular growth (27), implicating carbon assimilation to the cell wall. Additionally, diel random bar-code transposon-site sequencing (RB-TNseq) with *S. elongatus* determined that these processes were vital during the LD growth versus continuous light (28), consistent with our observations of carbon metabolism and cell wall synthesis in sync with the LD cycle.

We next examined other functions not clearly tied to metabolism, which could have preferential expression in the light. Notably, a subset of two-component system genes was found with high Light24 expression (Fig. S7). Closer inspection revealed that the majority of these preferentially expressed genes belong to chemotactic systems specifically expressed within developing hormogonia (29, 30). This finding suggests that light or the LD cycle influences the development of hormogonia. Indeed, when genes with experimentally validated functions in hormogonia (Table S6) were examined for preferential expression across the time-course series, the majority revealed significant upregulation within the Light24 time point (Fig. S8). Pronounced Light24 expression could indicate that motile hormogonium development and underlying transcriptional cascades are activated in response to constant light.

### Nucleotide metabolism and DNA repair during darkness 

Although most genes that were preferentially expressed in periods of dark remain poorly characterized, we were able to identify several functions that were significantly enriched and co-expressed in these time points. Elevated expression in the dark (mid-dark and/or pre-dawn) was most associated with purine metabolism, homologous recombination, and nucleotide metabolism (Fig. 4A). Notably, these functions were most enriched in both Pre-Dawn1 and Pre-Dawn2 time points (Fig. 4B; Fig. S7). By contrast, genes linked to these, or other core cellular processes, were not more highly expressed in any mid-dark time points. This finding raises questions about pre-dawn timing of gene expression and precise regulatory control during diel growth in multicellular cyanobacteria.

Prior studies have shown that circadian rhythm regulates the storage and conversion of sugars in *S. elongatus* (31), where specifically, RpaA regulates carbohydrate storage during the day and energy conversion at night. Separately, the transcriptional regulator cyAbrB was found to control the day-night shift in carbohydrate metabolism in *Synechocystis sp*. PCC 6803 (32). The diel transcriptome of *N. punctiforme* offered evidence that during darkness, stored sugars are primarily used to generate purines and nucleotide pools. Specifically, genes enriched during darkness are central to the pathways that convert nucleoside monophosphates to nucleotide bases (Fig. S9).

We next examined the individual genes that comprise the core cellular functions associated with predawn. Pronounced expression of *recR, recG, and recD* (Npun_F4120, Npun_R5567, Npun_AR019) was observed during pre-dawn time points (Fig. S7). RecD is the only component of the bacterial RecBCD complex encoded in the genome of *N. punctiforme*, and notably, it is a plasmid-encoded helicase. RecG is a multifunctional ATP-dependent DNA helicase, which is thought to contribute to chromosome repair, acting synergistically with the RuvABC complex (33). Interestingly, the diel transcriptome of *N. punctiforme* revealed that this single protein was enriched during the pre-dawn timepoint, which raises questions on the specific timing of DNA repair in filamentous cyanobacteria. The repair proteins were previously shown to be duplicated and highly conserved in several cyanobacterial genomes (34).

Two of six total proteins that can be components of the DpoIII complex were enriched in the pre-dawn transcriptome (Fig. S7): gamma/tau complex (Npun_F4123), and the alpha subunit (Npun_F5684). The gamma/tau subunit is predicted to promote processivity, through loading the beta clamp onto DNA (35). The alpha subunit holds the catalytic core of DpolII, wherein DNA synthesis proceeds (36). Taken together with the diel expression of repair proteins, these findings suggest that filamentous cyanobacteria may sequester chromosomal maintenance during darkness. Furthermore, given the necessity for damaged DNA to be repaired for efficient replication by DpoIII, we speculate that RecG-based repair may be timed synergistically with DpoIII expression. Indeed, the gamma/tau complex expression correlated with *recR* (*r =* 0.63), *recG* (*r* = 0.91), and *recD* (*r* = 0.90) expressions. By contrast, DpoIII alpha subunit expression negatively correlates with that of *recR* (*r =* −0.78), *recG* (*r* = −0.81), and *recD* (*r =* −0.75).

We next examined gene expression of repair proteins during the Dark-24 time point, which represents a perturbation of the diel cycle. Notably, a cluster of four DNA processing and repair genes was disproportionately expressed following this perturbation, comprising Dpo III α and δ, ssDNA exonuclease RecJ, and repair protein RecO (Fig. S7). Importantly, expression of these genes during Dark-24 was in sharp contrast with their comparatively low expression during the Light-24 perturbation, suggesting a specific response to darkness. Finally, many genes that were clustered with highest expression under dark conditions remain hypothetical/uncharacterized. Thus, it is unclear whether other cellular pathways are programmed to be upregulated in the dark.

### Transcription of circadian and clock-controlled proteins

While the global transcriptome appeared to partition primarily in response to light, a circadian shift for putative clock-controlled genes was observed during the pre-dawn/pre-dusk transitional time points. Cyanobacterial circadian rhythms are driven by diel oscillation in protein phosphorylation vs dephosphorylation (37), and as such, we did not expect diel cycling of expression for *kaiABC* core circadian genes (Fig. S10A). Notably, however, circadian clock-associated input-output proteins (*sasA, cikA*) and master regulators (*rpa,* and *rpaB*; Fig. S10B) exhibited cyclical expression peaking in the pre-dawn time points. Importantly, the GAF sensor CikA plays an intermediary role synchronizing the LD cycle with the circadian clock. The predicted CikA homolog in *N. punctiforme* (Npun_F1000) has high amino acid similarity (Table S3) to the canonical *Synechococcus elongatus* CikA, yet surprisingly, we did not observe elevated expression across any of the time points (Fig. S10B).

To predict functions of alternative kinases that may fulfill the CikA role within *N. punctiforme*, we identified 12 *cikA*-like genes encoded throughout the genome based on sequence similarity to cyanobacterial proteins containing CikA-like GAF and histidine kinase domains (Fig. S11A; Table S4). While these genes share sequence similarity with canonical CikA proteins, we sought to assess their expression across the entire global transcriptome. Revisiting the Pearson correlation analysis, we uncovered a subset of genes associated (r ≥ 0.75) with cell division, peptidoglycan biosynthesis, and intermediary genes linking central carbon metabolism with peptidoglycan biosynthesis (Table S2). Among these, a single GAF-like histidine kinase (Npun_R5149) correlated with five genes of interest: Npun_F0907 (*glmU*; precursor to peptidoglycan biosynthesis); Npun_F2411 (*murG*; peptidoglycan biosynthesis); Npun_F4453 (Class B PBP; cell wall synthesis); Npun_R4507 (*bacA*; cell wall lipid modification); and Npun_R4933 (*cdv1*; cell division). Notably, this putative CikA-like gene exhibited cyclical expression that was most pronounced during pre-dusk and pre-dawn time points (Fig. S11B).

Interestingly, another *N. punctiforme cikA*-like gene (Npun_R2903) showed diel-cycling expression. Despite not being as highly expressed as Npun_R5149, Npun_R2903 had consistently higher expression than the canonical *cikA* across time points (Npun_F1000; Fig. S11B). Moreover, hierarchical clustering of the associated genes described above (Fig. S11C) revealed similarity to Npun_R5149 and canonical *cikA* (Npun_F1000). Both alternative CikA-like, GAF-domain-containing histidine kinases had low amino acid similarity to canonical *S. elongatus* CikA (Npun_R5149 29.60%; Npun_R2903 26.10%; Table S3) and cyanobacterial CikA-like proteins (Npun_R5149 26.90%; Npun_R2904 29.90%; Table S4). Despite low sequence similarities to the annotated CikA locus (Npun_F1000), these other GAF-like histidine kinases (Npun_R5149 and Npun_R2903) showed higher and more cyclical expression as compared with the annotated *cikA* locus, perhaps fulfilling the role of a regulatory link between the LD cycle and the circadian clock. Indeed, when assessing the transcriptome for potentially co-expressed genes, Npun_R5149 correlated with the greatest number of genes as compared with Npun_R2903 and Npun_F1000 (Fig. S11D). Critically, canonical *cikA* (Npun_F1000) only significantly (≥0.75) correlated with one gene implicated in LD cell division (Npun_F5950, *ylmG:* nucleoid distribution), which contrasts with the other CikA-like histidine kinases (Table S5).

We next sought to further examine the domain architecture and phylogeny for this GAF-like kinase protein (Fig. S11E). It appears that Npun_R5149 and similar genes in other filamentous cyanobacteria may provide an orthologous function to CikA of unicellular cyanobacteria in establishing the circadian clock. Most notably, whereas all CikA-like homologs maintain one histidine kinase and ATPase domain, in stark contrast to Npun_F1000, other loci encode an array of multiple GAF domains, duplicated PAS and PAC signal sensors, and one or more receiver domains. The GAF-containing histidine kinases (GAF-HKs) of *N. punctiforme* show architectural complexity through fusion of extracellular sensory domains (CHASE-type), histidine kinase phosphotransfer domains (HPT), and incorporation of transmembrane sites. The subset of highly (and dynamically) expressed GAF-HK proteins (Npun_R5149, Npun_F6362, Npun_R6149, Npun_R2903) each has architectures comprising sensor and receiver domains in contrast with Npun_F1000 and the CikA of *S. elongatus* (Synpcc7942_0644).

Although circadian mechanisms have been explored extensively within *S. elongatus*, physiological evidence of circadian rhythm is lacking for *N. punctiforme* (38). Recent gene reporter experiments demonstrated circadian clock activity in the closely related filamentous cyanobacterium, *Anabaena* sp. PCC 7120—another model organism of cyanobacterial multicellularity (elsewhere known as *Nostoc* sp. PCC 7120; 39, 40). Circadian clock-controlled proteins identified within strain PCC 7120 share homology to several *N. punctiforme* genes with apparent clock-influenced expression (Fig. S12). While some of these homologs show transcriptional oscillation, separate studies suggest that circadian-associated gene expression can be uncoupled from circadian protein activity and vice versa (37, 41). Arbel-Goren and coauthors demonstrated that expression of the clock-controlled gene, *pecB*, was synchronized among individual vegetative cells along filaments, while heterocyst differentiation appeared to be regulated or gated by the clock (18). Future investigations may elucidate the mechanism by which circadian rhythm influences differentiation of heterocysts, and whether these factors affect the development of hormogonia or akinetes.

### Mobile genetic elements are dynamically expressed

**Fig 5:**
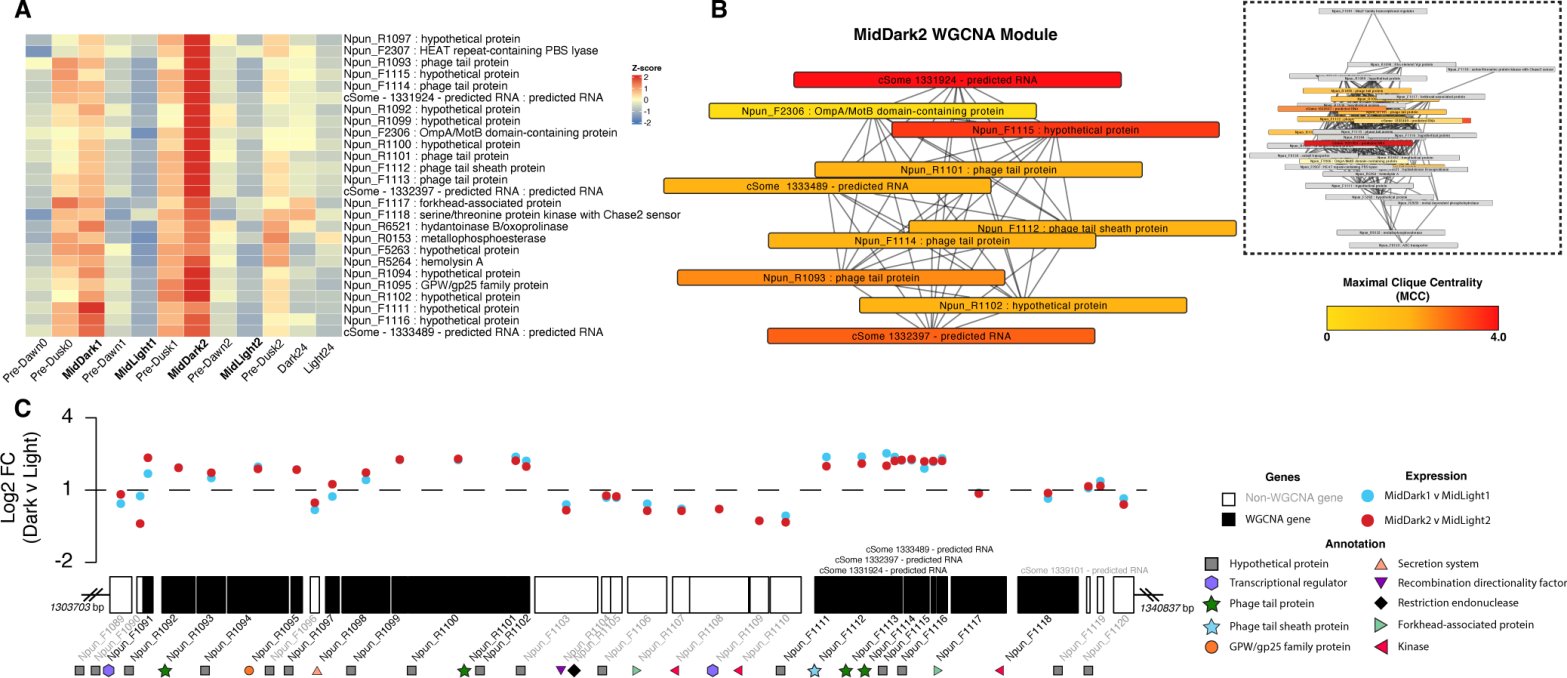
Analysis of MidDark2-associated putative prophage. (**A**) Heatmap of expression data from hierarchical clustering of the entire transcriptome, highlighting the MidDark2 phage gene cluster. Each gene’s expression is shown across each timepoint, with Z-score normalized expression (downregulated = blue, upregulated = red). (**B**) The weighted gene co-expression network analysis (WGCNA) module for MidDark2, including phage genes, distilled into the top 10 maximal clique centrality (MCC) nodes. The MCC scores indicating the centrality and importance of genes within the network are represented by a color gradient from low (yellow) to high (red). (**C**) Chromosomal region ranging from 1,303,703 bp to 1,340,837 bp encoding for phage-associated genes (black shading) and non-phage associated genes (white). Above the loci, log2 fold change (Log2 FC) is plotted for genes comparing MidDark1 vs MidLight1 (blue) and MidDark2 vs MidLight2 (red) time points. Annotations, where available, are provided for each locus.

*N. punctiforme* has been developed for several decades as a model organism for studying symbiosis and cellular differentiation in filamentous cyanobacteria. Yet, relatively little is known about the role of mobile genetic elements (MGE) in this organism. Many MGEs are well-characterized in pathobionts of humans and animals, where these systems play an important role in virulence (42–44). Moreover, in environmental and non-pathogenic microorganisms, the same MGE can often be identified via bioinformatic surveys; however, their functional significance and genetic activity are overlooked. We thus took advantage of our robust temporal data set to explore the dynamic subset of MGE in this model of filamentous cyanobacteria. Hierarchical clustering analysis of the global data set revealed several groups of genes with similar expression profiles across time points, including IS4 transposase genes, and two putative prophage elements (Fig. S13). Interestingly, a group of co-localized phage-like genes showed pronounced expression in the MidDark2 timepoint (Npun_F1091 - Npun_F1118), including five putative tail fiber proteins (Fig. 5A).

We next used network analysis to uncover separate host gene expression that correlated with expression of the putative prophage genes. Notably, the prophage locus associated with the MidDark2 time point was central to a weighted co-expressed gene network (Fig. 5B). This network was exclusively correlated with MidDark2 and stands out among other networks within the same time point (Fig. S4). Crucially, WGCNA confirmed that all of the phage tail genes are indeed co-expressed and revealed their broader network of associated genes; within this module, the 10 genes that represent the maximum clique centrality include four of the five phage structural genes (Fig. 5B). While this network encompasses a set of co-localized phage genes, other proximal genes show distinct expression profiles (Fig. 5C).

Within this gene neighborhood, we uncovered two apparent modes of expression, distinguished by putative structural genes versus genes with predicted recombination and regulation functions. The putative phage structural genes showed elevated expression (Npun_F1091-Npun_R1102 ; Npun_F1111-Npun_F1116) in MidDark2, whereas non-structural genes (Npun_F1103-Npun_R1110) were notably absent in the MidDark2 co-expression network. We hypothesize that the network of genes expressed during MidDark2—including phage tail, sheath, and a putative head morphogenesis GpW protein—is associated with virion assembly. These findings suggest that the temporal transcriptome captured a switching event, wherein a temperate virus may have been induced into its productive lytic cycle.

To assess whether this observation represents a stochastic even, or a persistent phenomenon, we carried out an additional growth experiment designed to target the same diel intervals (Fig. S14). Quantitative amplification of the locus, Npun_F1112 (phage tail sheath protein), confirmed that the putative phage gene was most expressed in the MidDark timepoint in both our original growth experiment and in the repeated experiment. Although median expression was highest in MidDark during the follow-up growth experiment, we did observe wide variation across replicates of the Pre-Dawn timepoint, wherein one replicate appeared to have higher expression than that of MidDark. Nonetheless, this independent assay supports the hypothesis put forth that structural genes found in a putative prophage element are preferentially expressed in the dark. Still, despite the expression of phage structural genes suggesting that the elements produce infective viral particles, they may instead represent replicative and integrative transposons that resemble MGE from other cyanobacterial mobilomes (45).

While cyanophage of globally abundant marine unicellular phytoplankton has been described in detail, the viruses of filamentous cyanobacteria remain relatively understudied. A collection of phage isolates from environmental blooms of *Nostoc* was previously described (46, 47), and more recently, three phage genomes were sequenced and contextualized (48). The putative temperate phage uncovered in this study is distinct and may represent a useful model to interrogate the host-phage dynamics and the mechanisms underpinning lysogeny in filamentous cyanobacteria.

### Hypermutation of conflict mitigation systems

**Fig 6:**
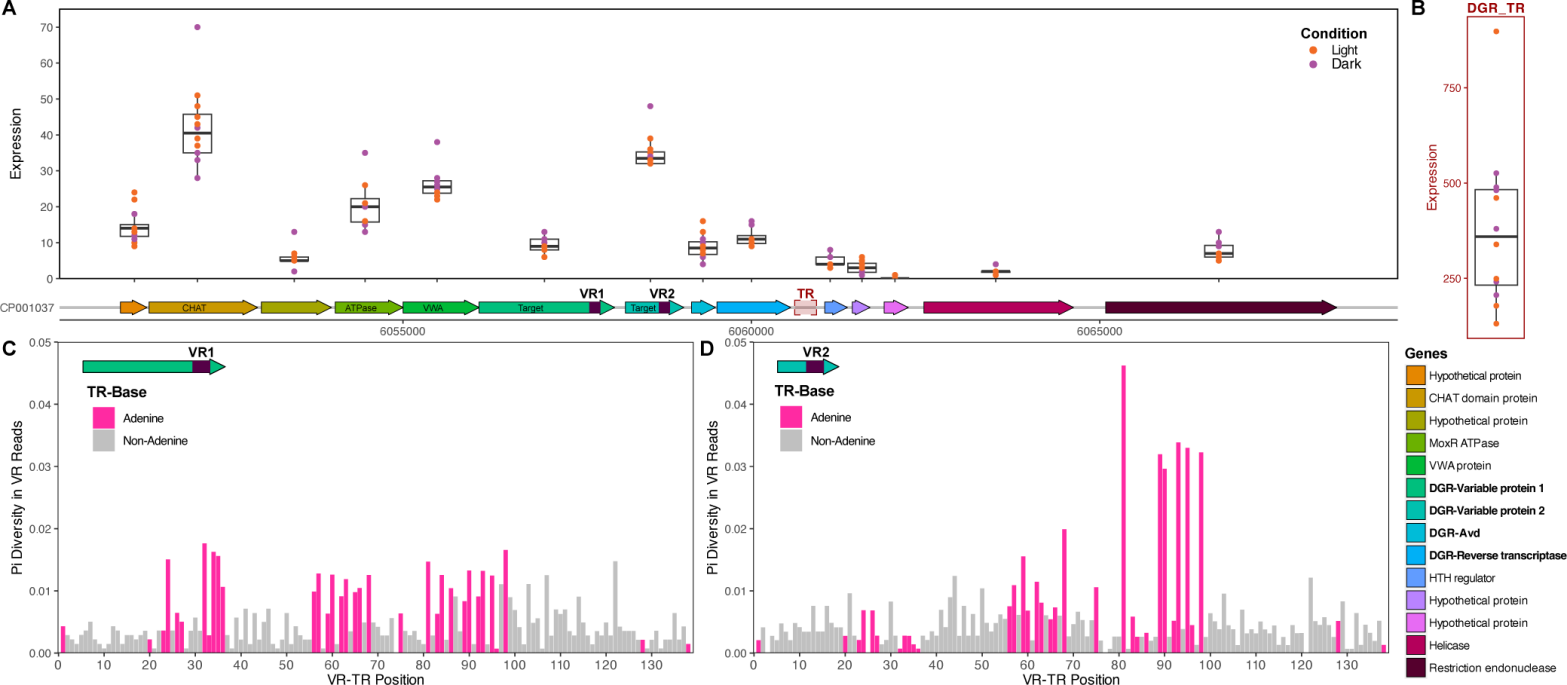
Diversity-generating retroelement (DGR) analysis. (**A**) Chromosomal region surrounding DGR locus, annotated with color-coded gene functions. Box plots represent the expression levels across 12 different time points, with each box plot displaying the median, quartiles, and range of expression values under light (orange) and dark (purple) conditions. Purple shaded boxes indicate the variable regions (VR) for DGR-variable proteins, and red shaded box indicates non-coding DGR template region (TR). (**B**) Box plot illustrating the expression levels of the DGR template region (DGR_TR) across the 12 time points (light = orange, dark = purple). (**C**) Nucleotide diversity (calculated as pi diversity) within Variable Region 1 (VR1) compared to the template repeat (TR). Adenine positions are indicated by pink shading. (**D**) Nucleotide diversity (calculated as pi diversity) within Variable Region 2 (VR2) compared with the template repeat (TR). Adenine positions are indicated by pink shading.

Diversity-generating retroelements (DGRs) are domesticated retrotransposons that drive targeted protein evolution and were recently highlighted as being prevalent within genomes of filamentous cyanobacteria (20). In most DGRs, a genomic locus encodes essential features, comprising a reverse transcriptase (RT) that complexes with an accessory variability determinant protein (Avd), template RNA repeat (TR) and cognate variable repeat (VR) coding site(s), which differ from the TR at adenines (i.e., adenosines in the template RNA). The putative DGR of *N. punctiforme* was previously described and has two target genes (20), but targeted mutagenesis has not been directly observed in these or other cyanobacterial cultures. The essential DGR components are all expressed (Fig. 6A), but one target gene appears disproportionately expressed in comparison with the second target. The known diversifying machinery, comprising RT and putative Avd, is consistently expressed across time points, though to a lesser degree than the neighboring genes.

Notably, TR-RNA appears to be very highly expressed (Fig. 6B), which is consistent with past observations (49, 50), yet unexpected for a domesticated model organism. This RNA serves a dual function—up- and downstream regions are predicted to form part of the ribonucleoprotein structure of a DGR complex (51), whereas the core TR sequence contains coding information transmitted to the target(s) via reverse transcription and retrohoming. In a previous study with the marine filamentous cyanobacterium, *Trichodesmium erythraeum*, multiple DGR loci were identified in which TR-RNA appeared highly expressed (49). However, an adenine mutation was not detected, which was attributed to long-term culture stability and loss of selective pressure for targeted diversification. By contrast, adenine mutation was observed in environmental metagenomes recruited to the *Trichodesmium* reference genome, suggesting that DGR mutation is stimulated in the wild (52). Thus, prior to this study, it was unclear whether DGR mutagenesis occurs or can be detected in lab-maintained isolates of filamentous cyanobacteria.

To detect hypermutation in the DGR target loci, we carried out high coverage whole-genome resequencing of *N. punctiforme* from the Dark24 timepoint, chosen as a perturbation of the natural LD cycle. Resequencing revealed dynamic adenine mutation in both target genes—while detectable in the variable locus of DGR target 1 (Npun_F4889; Fig. 6C), hypermutation is relatively higher in the variable locus of DGR target 2 (Npun_F4890; Fig. 6D). Despite uncovering a pattern consistent with adenine-specific mutation, Pi diversity was low in these loci—just above our limit of detection—suggesting that diversification is not elevated in the absence of specific environmental stress or stimuli. This is consistent with the expectation that under stable growth conditions, the cultures are not challenged with biological conflict (e.g., phage infection) or nutrient limitation. The low degree of targeted mutation is also consistent with low expression of the diversification machinery, RT, and Avd. It is unclear if the perturbation to the LD cycle influences hypermutation, or if hypermutation is constitutive at low levels under general laboratory conditions. Future experiments designed to compare hypermutation between LD and LL cycles can address this question. These findings also raise the question of whether regulation of hypermutation is decoupled from the expression of the target genes themselves.  

While little is known about the functional role of hypermutation target genes of cyanobacterial DGRs, they have been recently hypothesized to coordinate immunity or defense as part of multi-protein conflict mitigation systems (53). To determine whether co-localized genes in the DGR neighborhood were co-expressed, we conducted a global Pearson correlation analysis across pairwise sets of genes (Table S7). This uncovered a group of apparently correlated genes, comprising a CHAT domain-containing protein (Npun_F4885), a MoxR ATPase (Npun_F4887), a VWA domain-containing protein (Npun_F4888), and DGR target 2 (Npun_F4890). Moreover, expression of DGR-RT (Npun_F4892) appears correlated with a downstream restriction endonuclease (Npun_F4897). It is perhaps surprising that putative Avd and DGR-RT were not correlate;, however, this is likely a result of their overall low expression across the time series.

The co-occurrence of MoxR and VWA proteins (known as STAND-family ATPases) with DGR hypervariable targets was proposed to play a role in adaptive defense by diversifying antigen sensors, thereby mitigating conflicts, such as phage infection (53). Our findings offer new experimental evidence that co-localized STAND ATPase and DGR target genes are indeed co-expressed. As such, targeted hypermutation of putative conflict systems likely confers a fitness advantage in wild filamentous cyanobacteria. While the specific cellular function of these multipartite systems is somewhat elusive, DGRs could offer phenotypic heterogeneity and subpopulation stability during biological conflict.

### Concluding remarks

Our study offers a comprehensive view of diel expression dynamics in *Nostoc punctiforme*. The results highlight a clear shift in day-to-night expression of genes involved in a myriad of cellular processes—notably, central carbon metabolism, nucleotide and amino acid conversion, and DNA repair. A combined workflow of clustering, network, and sequence diversity analyses revealed a dynamic mobilome consisting of transposons, prophage, and retroelements that drive hypermutation. Despite long-term domestication under continuous light and stable culture conditions, this multicellular cyanobacterium still partitions its gene expression into light- and dark-associated processes. Co-occurring sugar metabolism and peptidoglycan biosynthesis, along with genes for hormogonium development and motility, were preferentially expressed in the light. Conversely, dark conditions led to the preferential expression of chromosome repair and nucleotide metabolism pathways. And importantly, several putative protein kinases were linked to diel oscillations in expression, despite key differences in sequence and structure relative to the canonical input kinase, CikA. These findings suggest that gene paralog expansion in multicellular cyanobacteria may have evolved input proteins that establish clock synchronicity, which are distinct from those in unicellular cyanobacteria.

Throughout this temporal study, several mobile genetic elements, including an array of transposons and a putative prophage, showed variable expression across the time series experiment. Additionally, we confirmed the dynamic expression of diversity-generating retroelements, accompanied by adenine-specific hypermutation in target genes. Collectively, these findings offer significant insights into the regulatory complexity and genotypic plasticity of this model organism.

Some limitations arose when contextualizing the transcriptome of *N. punctiforme*—especially where detailed information on diel regulation is available for unicellular cyanobacteria that may not apply in multicellular counterparts. For example, while *Synechococcus* spp. members exhibit markedly lower levels of mRNA at night (41, 54), *Anabaena* spp. maintain constant mRNA concentrations throughout light-dark cycles (39), exemplifying key differences in expression between unicellular and multicellular cyanobacteria. Furthermore, the core molecular mechanism of circadian rhythm is a proteinaceous phenomenon that involves oscillatation between protein phosphorylation and dephosphorylation states. Input to the circadian clock is synchronized by proteins that respond to diel shifts in cellular metabolism and the internal redox state, while clock output leads to diel expression dynamics for many genes under global control. Instances of delayed circadian control, likely due to post-transcriptional regulation (37), further complicate interpretation of expression data. As such, future efforts will benefit from the deconvolution of multicellular circadian and diel processes, both in terms of global transcription and protein (e.g., phosphorylation) states.

The diel transcriptome of *N. punctiforme* provides a new benchmark for assessing temporal changes in gene expression in filamentous cyanobacteria. Looking ahead, experimental efforts with *N. punctiforme* and other filamentous cyanobacteria should aim to incorporate the diel cycle while assessing the development of specialized cells and the process of nitrogen fixation. This study reveals natural diel rhythms in core cellular processes of a model filamentous cyanobacteria, which can inform future efforts to better understand the regulatory underpinnings of multicellularity and adaptation in these organisms.

## MATERIALS AND METHODS

### Bacterial culture conditions

*N. punctiforme* PCC 73102 (ATCC 29133) was grown at 28°C under constant shaking conditions (130 rpm) within Allen and Arnon standard growth media, diluted fourfold (A&A/4) but not amended with combined nitrogen, as to avoid an unintended NH_4_-step-down effect (55). Cultures were grown under constant light (40–50 µmol photons m^−2^s^−1^) for 2 weeks to accumulate biomass prior to partitioning into replicate cultures equivalent to ~47 µg chlorophyll a. diel entrainment was simulated via a 12-h Light/12-h Dark (LD) growth regime, and triplicate cultures were collected per examined timepoint. To collect the final two samples (Light24 and Dark24), the 12/12 LD regime was disrupted 192 h into the experiment (~Pre-Dusk2). At this time, a 24-h extended LL incubation was performed, with Dark24-designated replicate cultures covered in tin foil to occlude light (24-h DD).

### Sample collection and nucleic acid extraction

Sample collection and nucleic acid extraction were performed as previously described (30, 56). Briefly, cultures were harvested via centrifugation and bacterial pellet cryopreservation at −80°C. Frozen pellets were subjected to lysis via bead-beating, and total RNA was precipitated with lithium chloride precipitation. RNA was further purified with the QIAGEN RNeasy Plus Mini Kit and on-column DNase treatment (QIAGEN RNase-Free DNase Set), per manufacturer’s instructions.

Genomic DNA was similarly extracted from frozen bacterial pellets. Pellets were subjected to initial lysis with lysozyme and bead-beating; total DNA was isolated following Zymo Research Quick-DNA Miniprep Plus Kit manufacturer instructions.

### Transcriptome sequencing

Enrichment of bacterial mRNA was performed using the NEBNext rRNA Depletion Kit (Bacteria) and RNA sample purification beads. Non-directional cDNA libraries were synthesized using the Invitrogen SuperScript IV First-Strand Synthesis System. Library preparation and sequencing were performed by the Keck Sequencing Facility at the Marine Biological Laboratory. Briefly, sequencing libraries were prepared using the Tecan Life Sciences Ovation Ultralow V2 DNA-Seq Library Preparation Kit, followed by two Illumina NextSeq 500 sequencing runs (Fig. 1A: Stages I and II; Stages III and IV). Library amplification was carried out with 10 to 14 cycles (10 cycles for replicate i, and 14 cycles for replicates ii and iii across all time points). Single-end reads were generated using the Illumina NextSeq 500/550 High Output Kit v2.5 (150 cycles).

### Whole genome sequencing

One biological replicate of genomic DNA extracted from the Dark24 time point was selected for whole genome sequencing. Library preparation was conducted as above (i.e., for RNA seq libraries) but with seven cycles of library amplification. Sequencing was performed by the Keck Sequencing Facility at the Marine Biological Laboratory library. Paired-end reads were generated using the Illumina NextSeq 500/550 High Output Kit v2.5 (300 cycles).

### Bioinformatics

Rockhopper was used with default parameters for the alignment, assembly, and normalization of sequencing data (57, 58). An average of 19,993,756 reads were mapped to the *N. punctiforme* genome (NCBI RefSeq assembly GCF_000020025.1) for all replicates and time points. Visualizations of RNAseq data (Spearman’s correlations and principal component analysis) were calculated within R for upper quartile normalized gene counts of all samples. The bioinformatic workflow is presented within Fig. S3, wherein the global transcriptome was first filtered to remove low expression (i.e., <10 expression in all time points). Parsed expression values were Z-score normalized per gene before hierarchical clustering (distances: Euclidean; linkaging: complete; R package ComplexHeatmap v2.16.0 [59, 60]).

WGCNA derived from all 12 experimental transcriptomes were generated with R package WGCNA v1.72-5 (61, 62). Pearson correlation values were calculated to examine co-expressed gene networks (“modules”) correlations with time points. Cytoscape v3.9.1 (63) was used to visualize modules, and cytoHubba v0.1 (64) was used to calculate maximal clique centrality (MCC). Broadly, “day” encompassed Pre-Dusk, MidLight, and Light24 time points, whereas “night” referred to Pre-Dawn, MidDark, and Dark24 time points.

We examined the “core” diel (LD) entrainment transcriptomes (Fig. 1A, Stages II and III) for diel-variable expression. Genes having consistently low expression were filtered (as previously), prior to an analysis of variance (ANOVA) of the four states (MidDark, Pre-Dawn, MidLight, Pre-Dusk) in duplicate for the core eight transcriptomes, with Benjamini-Hochberg (BH) correction. Hierarchical clustering of significant genes (BH-adjusted *P* < 0.05) revealed distinct “day” (Pre-Dusk and MidLight) and “night” (Pre-Dawn and MidDark) associated partitioning into two major clusters. These “day” and “night” associated genes, in combination with the previously identified WGCNA modules most-correlated with each timepoint, were submitted for gene set enrichment analysis (GSEA; R package clusterProfiler v4.8.3 (65, 66) and *N. punctiforme* KEGG entries to identify “day” and “night” associated cellular processes.

Reciprocal BLASTn (v2.12.0+; default settings) analyses of clock-controlled *Nostoc* sp. PCC 7120 (NCBI RefSeq assembly GCF_000009705.1) genes (39) and *N. punctiforme* coding sequences were performed to identifyhomologss, with potential cyclical expression visualized within KEGG pathway-unique polar plots.

*Synechococcus elongatus* PCC 7942 CikA (Synpcc7942_0644; UniProt entry Q9KHI5) GAF region (residues 184–338) and histidine kinase domain (residues 390–611) were separately submitted to two BLASTp searches restricted to NCBI-available cyanobacteriota (v2.12.0+; Taxonomy ID 1117; default settings). Clustal Omega (v1.2.3; default settings) was used to align proteins containing both domains, generating a consensus sequence that was used for a tBLASTn (v2.12.0+; default settings) search of *N. punctiforme* genome, with a 50% query coverage threshold for determining CikA-like histidine kinases.

Whole genomic sequencing reads were aligned to the *N. punctiforme* genome (NCBI RefSeq assembly GCF_000020025.1) using bwa-mem2 (67). Mapping file sorting and coverage were completed with Samtools v1.17 (68). A custom python script was developed to calculate the pi diversity of the predicted DGR hypermutable region.

### RT-qPCR

A separate diel experiment was performed for reverse transcription-quantitative PCR (RT-qPCR) examination of *N. punctiforme* expression. Eighteen replicate flasks of AA/4 were inoculated with biomass equivalent to ~51 µg chlorophyll ɑ before incubation at repeated growth conditions (12 L/12 D cycle, light intensity, temperature, shaking conditions). Cultures were grown for 47.5 h prior to sample collection (as previously described) for the Pre-Dawn timepoint. Subsequent samples were collected every 6.5 h for the MidLight, Pre-Dusk, and Mid-Dark time points, yielding triplicate replicates per timepoint. To collect the final two samples (Light24 and Dark24), the 12/12 LD regime was disrupted 67 h into the experiment (~MidDark). At this time, a 24-h extended LL incubation was performed, with Dark24 designated replicate cultures covered in tin foil to occlude light (24-h DD).

RT-qPCR samples were collected and processed similarly to the RNAseq workflow. The Applied Biosystems QuantStudio 5 Real-Time PCR System and the NEB Luna Universal qPCR Master Mix were used to quantify selected genes’ expressions. In addition to the repeated diel growth samples (*n* = 18), the last 18 RNAseq samples were submitted for RT-qPCR (Fig. 1A; Stages III and IV). Normalization was performed with *rnpB* (Npun_r018; RNA component of RNaseP; F 5′-TAAGAGCGCACCAGCAGTAT-3′ and R 5′-CATTGAGCGGAACTGGTAAA-3′). Chosen experimental genes were *murA* (Npun_R5719; UDP-N-acetylglucosamine 1-carboxyvinyltransferase; F 5′-CCTCGCACATGCAAGTCAAC-3′ and R 5′-TCGCCATTGGAGCCATTCTT-3′); *psbF* (Npun_F5552; photosystem II cytochrome b559 subunit beta; F 5′-AGCGGCAATAACATCAATCAACC-3′ and R 5′-AAATTGCATTGCGGCGATCG-3′); and a phage tail sheath protein gene (Npun_F1112; F 5′-TACTTTGCCCGTCCCAACTC-3′ and R 5′-AACGACCGCCACCATTCATA-3′). Standard curve and absolute quantification calculations were performed within Applied Biosystems Design & Analysis Software v2.7.0.

## Data Availability

Transcriptome raw data, metadata, and gene counts were uploaded to the GEO database with the accession number GSE275682. WGSeq raw reads were submitted to the SRA database under accession number SRR30545090 and will become publicly available once accession formalization resumes. R and Python scripts are available on GitHub at https://github.com/The-Paul-Lab/NpunDielExpressionDynamics.git.

## SUPPLEMENTAL MATERIAL

Supplemental Figures mbio.03779-24-s0001.pdf Figures S1 to S14.

Supplemental Tables mbio.03779-24-s0002.pdf Tables S1 to S7.

ASM does not own the copyrights to Supplemental Material that may be linked to, or accessed through, an article. The authors have granted ASM a non-exclusive, world-wide license to publish the Supplemental Material files. Please contact the corresponding author directly for reuse.
