# Supplementary material for: Diel expression dynamics in filamentous cyanobacteria
Source: mBio. 2025 Nov 18;16(12):e03779-24. doi: 10.1128/mbio.03779-24 (PMC12691664; doi:10.1128/mbio.03779-24)
Supplement: Supplemental Figures — Figures S1 to S14. [file mbio.03779-24-s0001.pdf]

Supplemental Figures

Diel expression dynamics in filamentous cyanobacteria

Sarah J. Kennedy[1], Douglas D. Risser[2], Blair G. Paul[1,*]

[1]Bay Paul Center, Marine Biological Laboratory, Woods Hole, MA 02543

[2]Department of Biology, University of Colorado Colorado Springs, Colorado Springs, Colorado, USA

Figs. S1 to S14

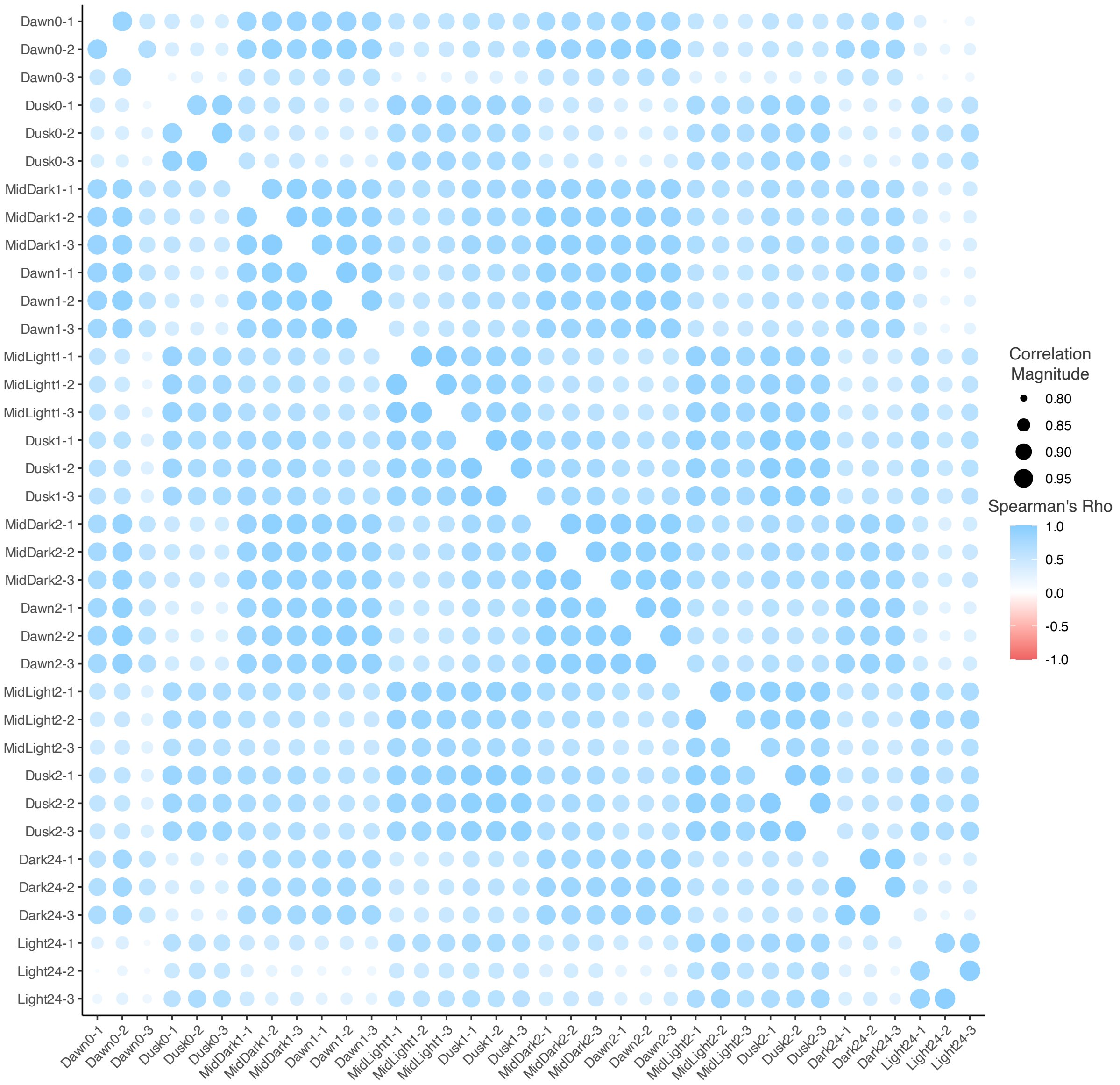

Supplemental Figure S1. Spearman correlation analysis of normalized expression counts.
Spearman correlation plot of normalized expression counts for all samples across the time-course experiment. Each circle represents the Spearman correlation coefficient between pairs of samples, with values ranging from -1.0 (perfect negative correlation) to 1.0 (perfect positive correlation). The size of each circle is proportional to the amount of transcriptional data. The heatmap is color-coded, with blue indicating positive correlations and red indicating negative correlations. Timepoints include Pre-Dawn, MidDark, Pre-Dusk, MidLight, Dark24, and Light24, with triplicate samples for each condition.

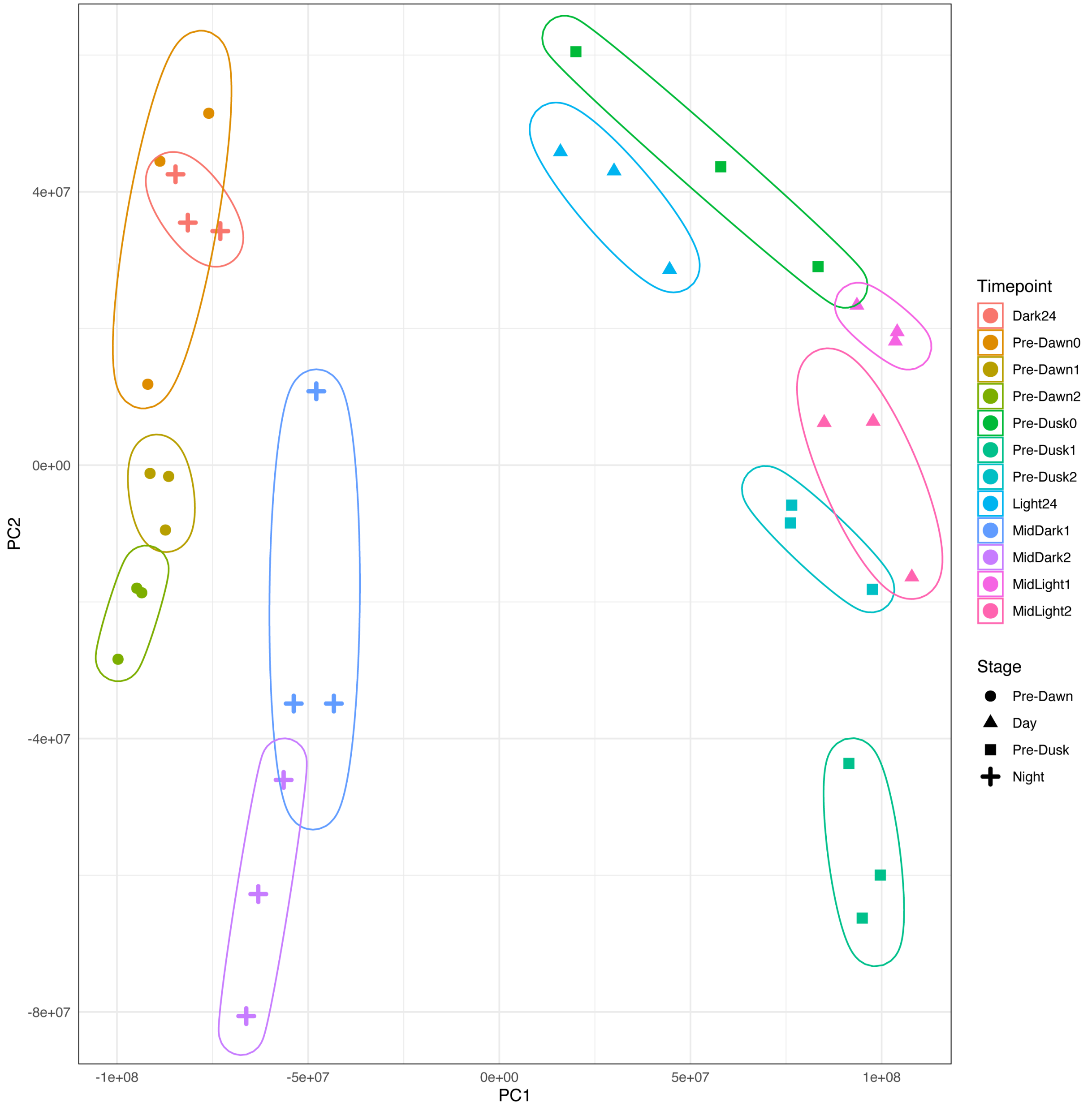

Supplemental Figure S2. Principal component analysis of normalized expression values.
Principal Component Analysis (PCA) of the normalized expression values for all 36 samples across the time-course experiment. Expression distribution by sample is shown for the first two principal components (PC1 and PC2). Each point represents a distinct sample, and timepoints are shown as separate colors. Shapes indicate the stages: circles for Pre-Dawn, squares for Pre-Dusk, triangles for Day, and plus-signs for Night.

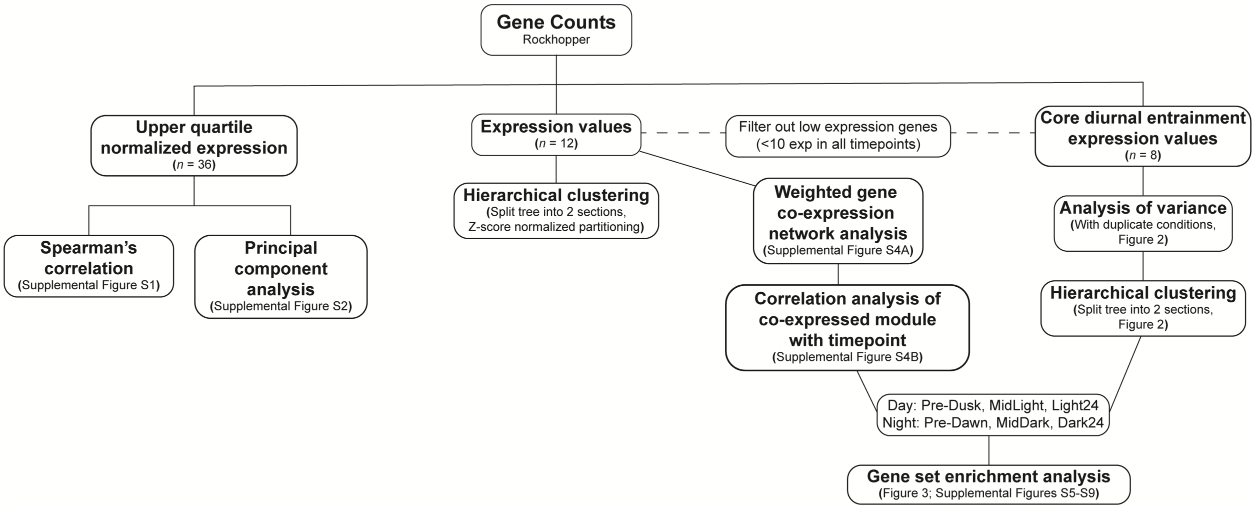

Supplemental Figure S3. Transcriptional data processing workflow.
Flowchart outlining the transcriptional data processing steps. Upper quartile normalized expression for all 36 samples was used for Spearman's correlation and principal component analysis. Low expression genes (less than 10 expression in all timepoints) were filtered out before subsequent analyses.

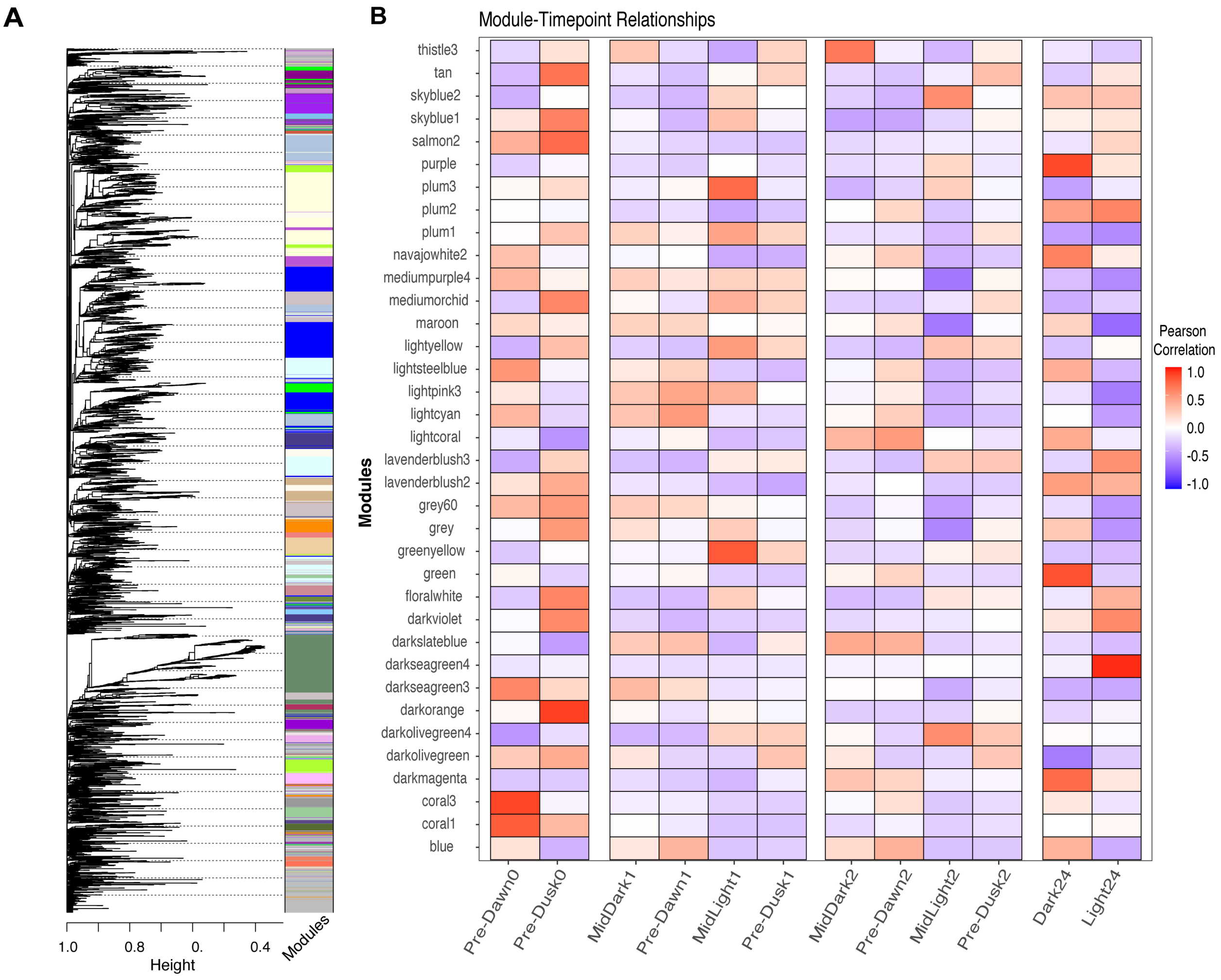

Supplemental Figure S4. Weighted gene co-expression network analysis.
(A) Clustering of the transcriptome into modules of co-expressed genes. Each branch represents a module containing genes with similar expression patterns across the 12 timepoints. (B) Pearson correlation matrix showing the relationships between the 12 timepoints and the modules of co-expressed genes. The correlation coefficients are color-coded, with blue indicating negative correlations and red indicating positive correlations.

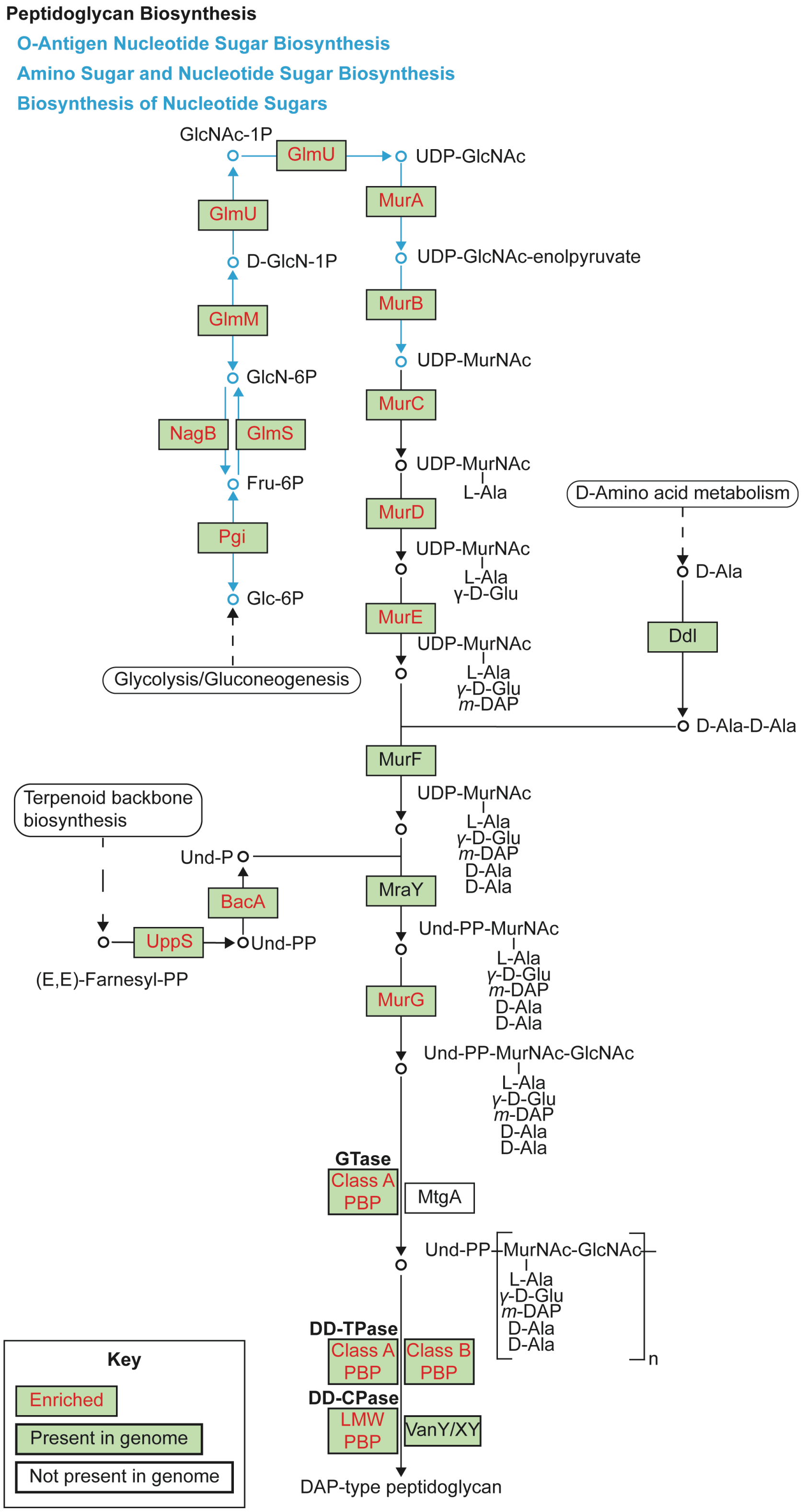

Supplemental Figure S5. Convergence of KEGG pathways most enriched in light.
The four KEGG pathways most enriched during light conditions are Peptidoglycan Biosynthesis, O-Antigen Nucleotide Sugar Biosynthesis, Amino Sugar and Nucleotide Sugar Biosynthesis, and Biosynthesis of Nucleotide Sugars. Enriched genes are presented in green boxes with red text, genes encoded in the Nostoc punctiforme but not enriched are presented in green boxes with black text, and genes not encoded in the N. punctiforme genome are in white unshaded boxes. Blue arrows indicate the biosynthetic pathways converging with peptidoglycan biosynthesis pathway (black arrows).

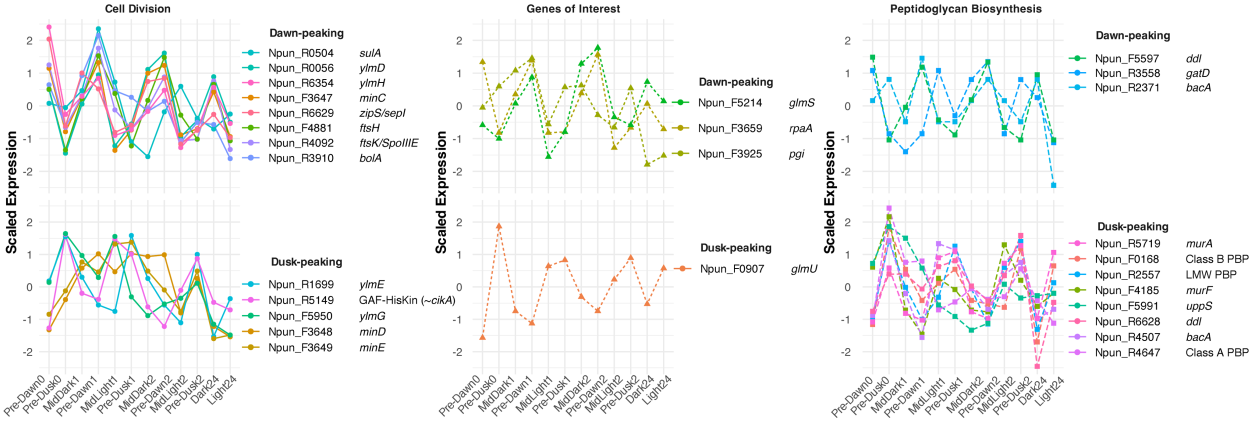

Supplemental Figure S6. Transcriptional convergence of cell division, sugar metabolism, and peptidoglycan biosynthesis.
Z-score normalized expression of genes involved in cell division, sugar metabolism, and peptidoglycan biosynthesis across different diel states. Expression values within each category are distinguished between dawn- and dusk-peaking patterns.

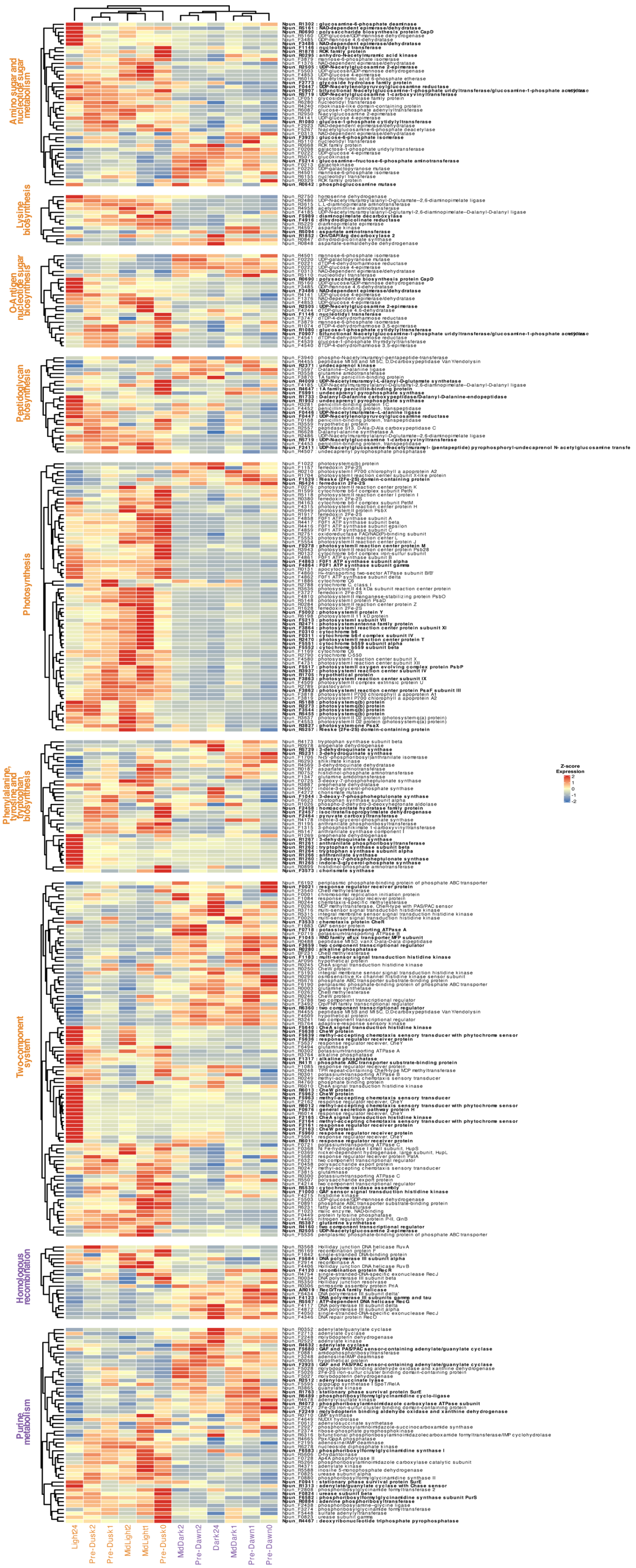

Supplemental Figure S7. Detailed Enriched KEGG Processes.
Hierarchical clustering (Euclidean distance, complete linkage) and z-score normalization (blue = downregulated, red = upregulated) of gene expression data highlight the light-associated (orange) and dark-associated (purple) enriched KEGG pathways. Enriched genes are bolded.

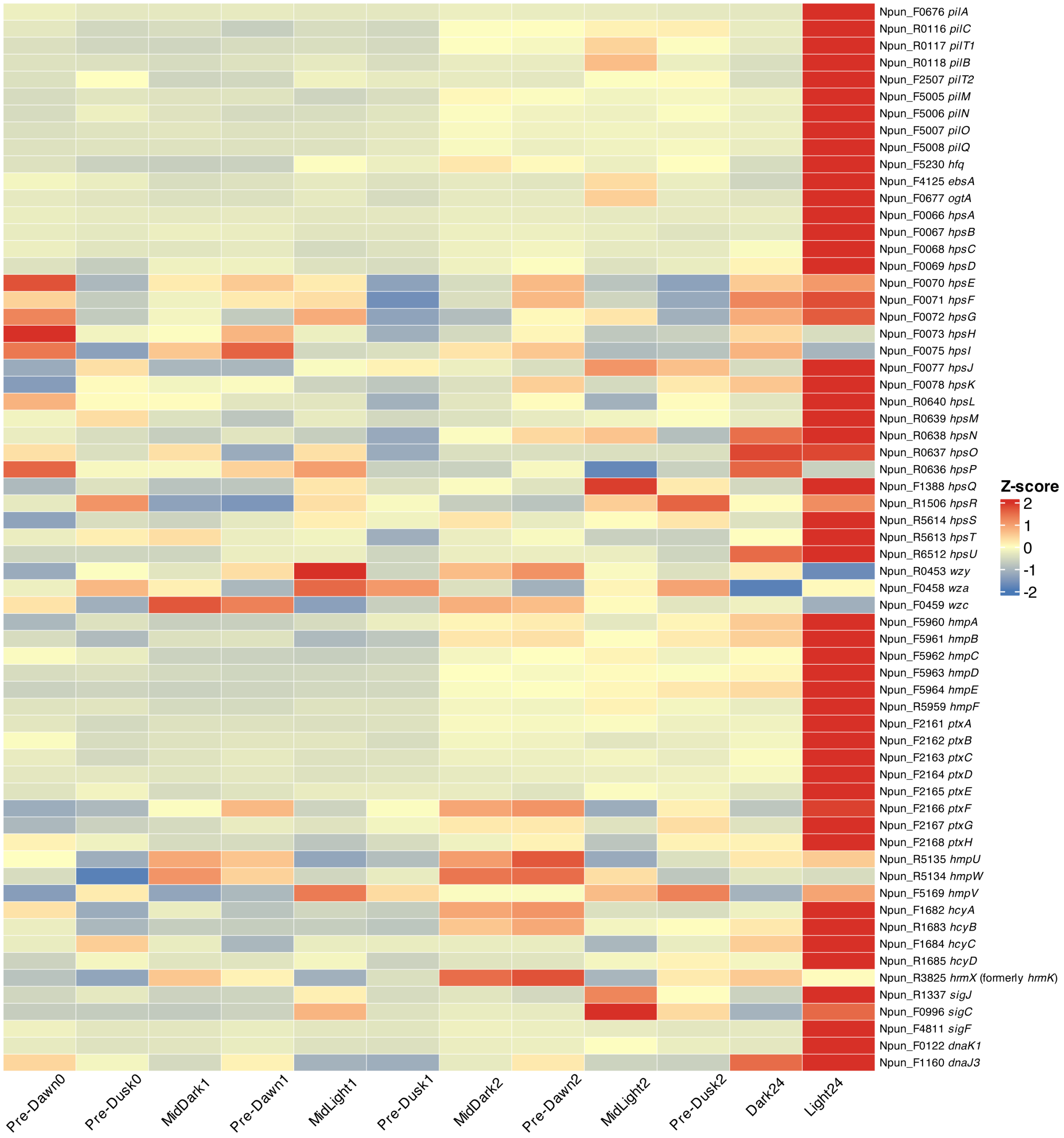

Supplemental Figure S8. Expression of hormogonium-associated genes.
Z-score normalized expression heatmap for all hormogonium-associated genes listed in Supplemental Table S6. Each row represents a gene, and each column represents a timepoint. The color gradient indicates the directional level of expression, with blue representing downregulation and red representing upregulation.

Nucleotide Metabolism and Purine Metabolism

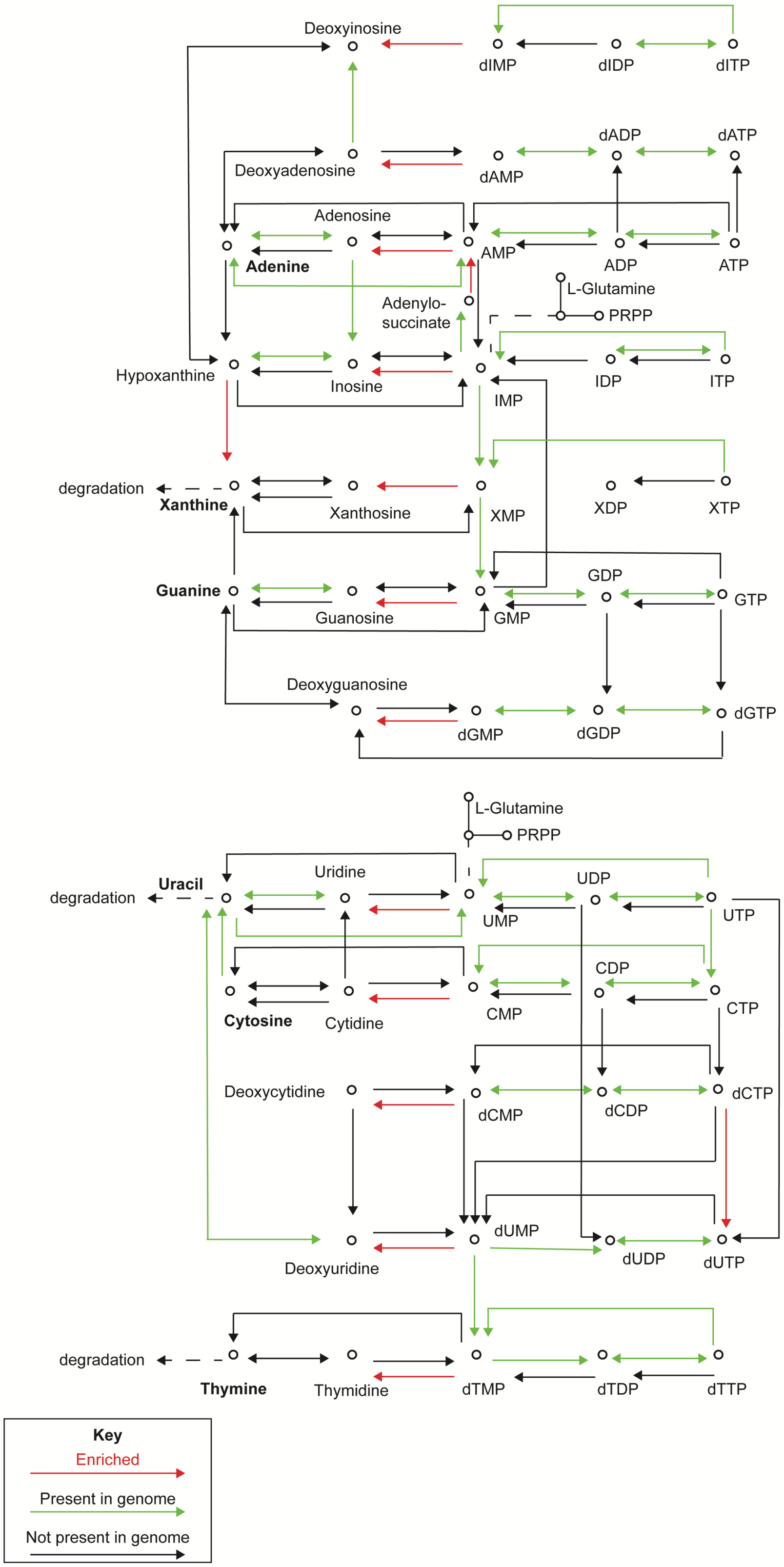

Supplemental Figure S9. Dark-associated nucleotide and purine metabolism pathways. Dark-associated pathways involved in nucleotide and purine metabolism, with red arrows indicating enriched genes, green arrows indicating pathways encoded in the genome but not enriched, and black arrows representing pathways not encoded in the genome.

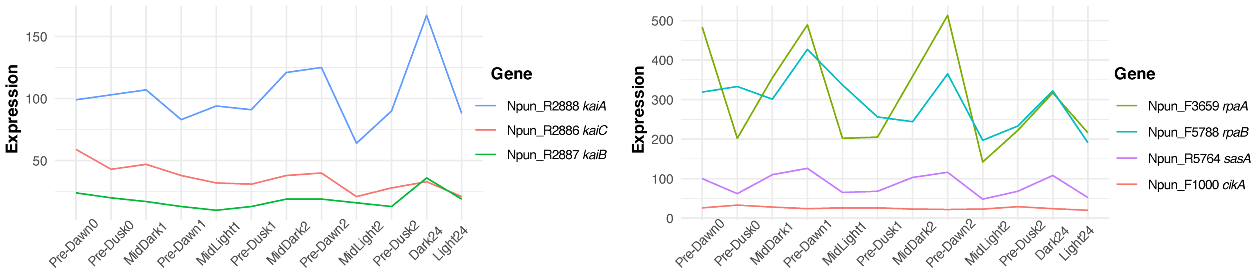

Supplemental Figure S10. Putative circadian gene expression.
(A) Expression levels of core Kai-protein clock genes (kaiA, kaiB, kaiC) and (B) non-core (rpaA, rpaB, sasA, cikA) across the time-course experiment.

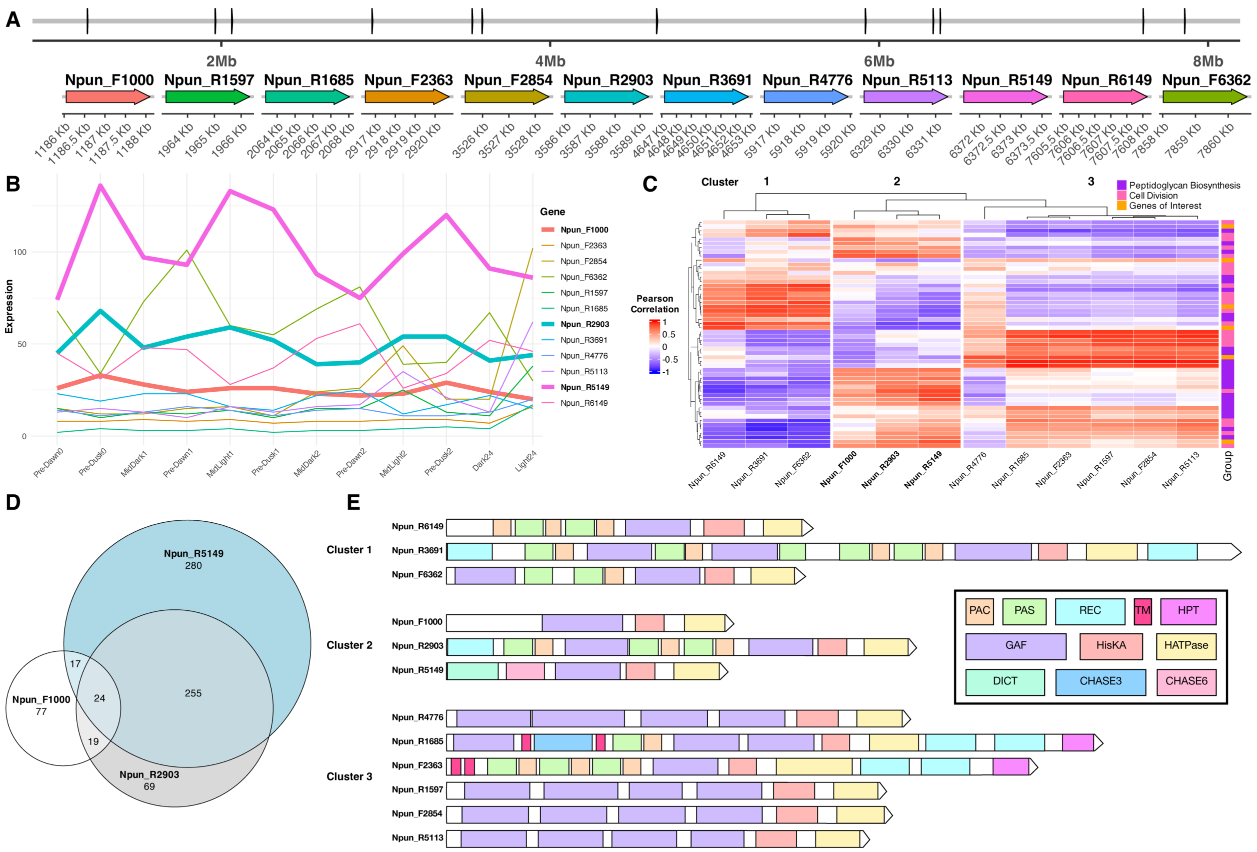

Supplemental Figure S11. N. punctiforme CikA-like paralogs containing GAF and histidine kinase domains.
(A) Genomic loci and nucleotide lengths of CikA-like paralogous genes in N. punctiforme. (B) Expression levels of CikA-like genes across the time-course experiment. (C) Hierarchical clustering (Euclidean distance, complete linkage) of Pearson correlations between CikA-like gene expression and selected genes within peptidoglycan biosynthesis (purple), cell division (pink), and key genes linking these functions (orange). The heatmap uses a blue-red gradient to indicate correlation values, with blue representing negative correlations and red representing positive correlations. (D) Euler diagram showing the distribution of global transcriptome genes with ≥0.75 correlation to the three CikA-like genes. (E) Domain architecture of the three clusters of 12 total CikA-like histidine kinases, highlighting the following domains: PAS (Per-Arnt-Sim), GAF (cGMP-specific phosphodiesterases, adenylyl cyclases, and FhlA), HisKA (Histidine Kinase A), HATPase (Histidine Kinase-Like ATPases), REC (Receiver domain), DICT (Diguanylate Cyclase domain), CHASE6 (Cyclase/Histidine kinase Associated Sensor domain 6), CHASE3 (Cyclase/Histidine kinase Associated Sensor domain 3), TM (Transmembrane domain), and HPT (Histidine Phosphotransfer domain).

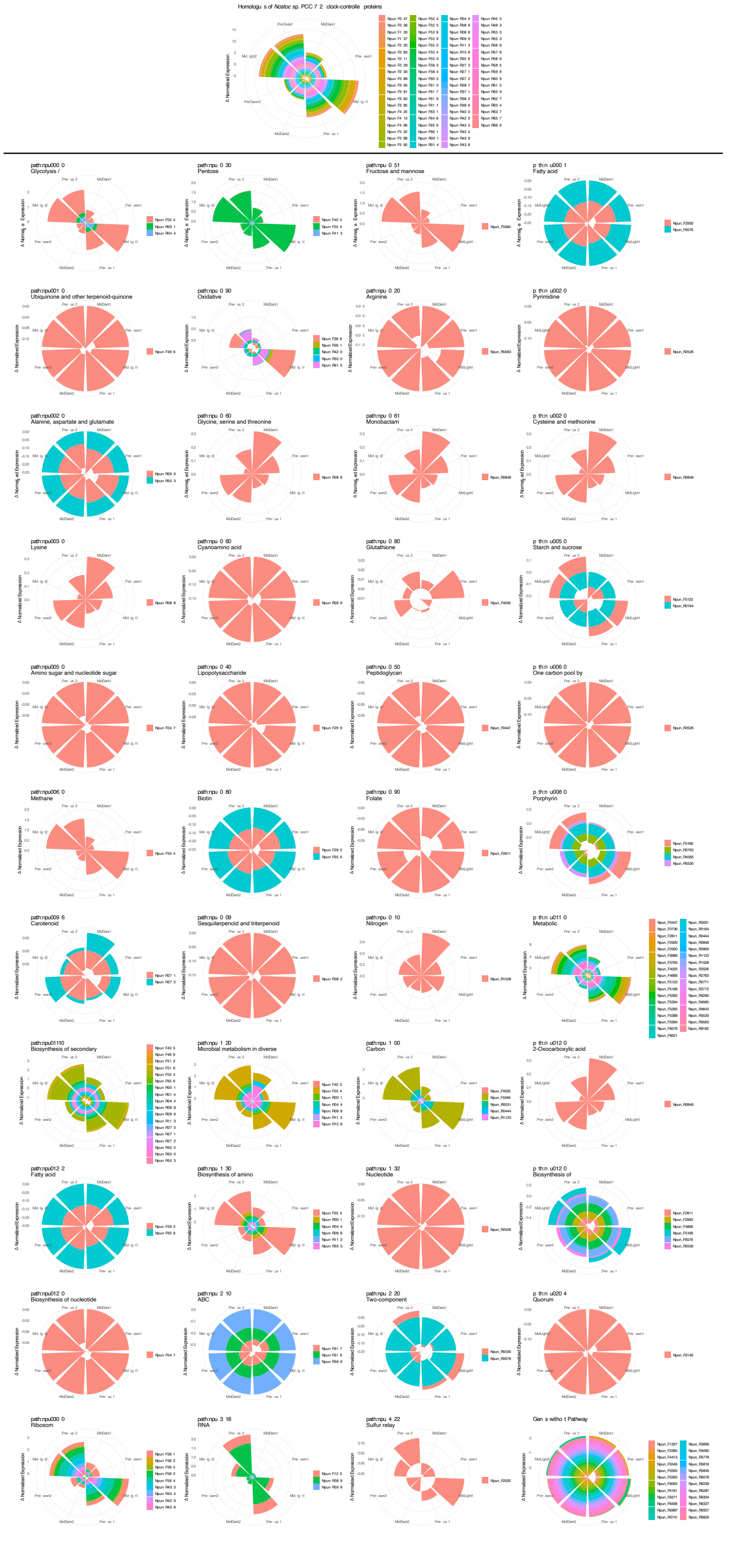

Supplemental Figure S12. Polar plots of clock-controlled protein homolog transcription.
Polar plots constructed by z-score normalizing each timepoint's transcriptome data for N. punctiforme genes homologous to Nostoc PCC 7120 experimentally-determined circadian clock-controlled proteins. (A) Polar plots excluding two highly expressed genes, cpcB (Npun_F5289) and cpcA (Npun_F5290), to maintain scale integrity. (B) Polar plots partitioned by KEGG pathways, showing the transcriptional patterns of clock-controlled protein homologues across diel states.

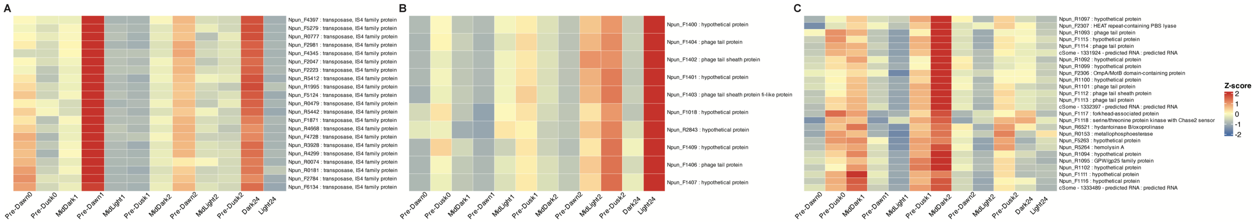

Supplemental Figure S13. Dynamically expressed mobile genetic elements.
Z-score normalized expression heatmaps of three dynamically expressed mobile elements across the time-course experiment, identified from the hierarchically clustered (Euclidean distance, complete linkage) transcriptome.
(A) Pre-Dawn1 + Dark24 cluster of IS4 family transposases. (B) Light24 cluster of phage proteins. (C) MidDark2 cluster of phage proteins. Heatmaps show expression patterns across the timepoints, with blue indicating downregulation and red indicating upregulation.

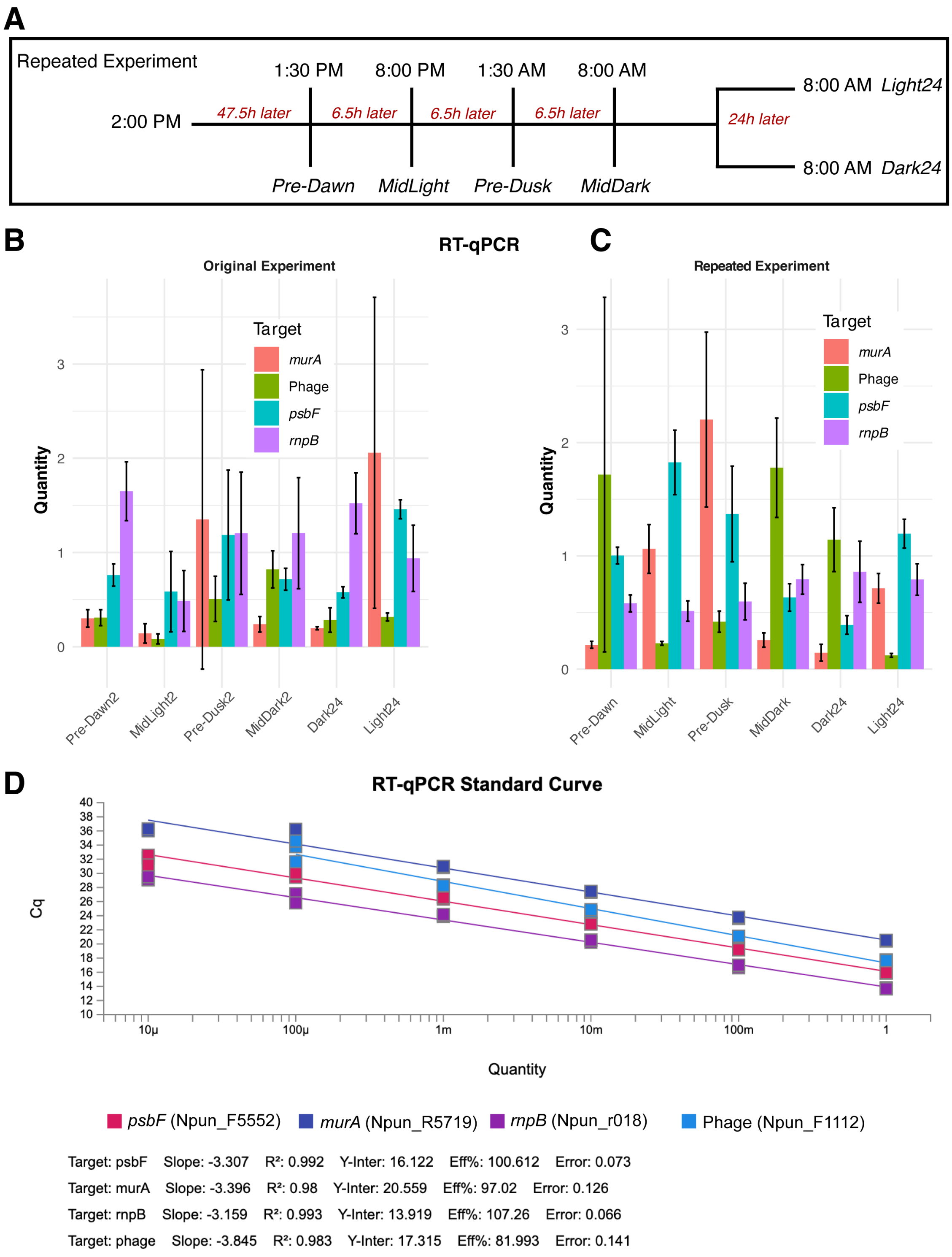

Supplemental Figure S14. Reverse transcription quantitative PCR analysis of gene expression.
(A) Repeated diel experiment collecting samples at Pre-Dawn, MidLight, Pre-Dusk, MidDark, and Light24 + Dark24 timepoints. (B) RT-qPCR absolute quantification of murA (DP-N-acetylglucosamine enolpyruvyl transferase), psbF (Cytochrome b559 beta subunit), and MidDark2 phage tail sheath protein, normalized to rnpB (RNase P RNA gene), for original experimental samples (Stages III and IV). (C) RT-qPCR absolute quantification for repeated experimental samples. (D) Standard curve for the RT-qPCR.
