# Supplementary material for: Diel expression dynamics in filamentous cyanobacteria
Source: mBio. 2025 Nov 18;16(12):e03779-24. doi: 10.1128/mbio.03779-24 (PMC12691664; doi:10.1128/mbio.03779-24)
Supplement: Supplemental Tables — Tables S1 to S7. [file mbio.03779-24-s0002.pdf]

Supplemental Table S1. Correlations of sugar metabolism and peptidoglycan biosynthesis gene expressions.

Gene1	Gene2	CorrelationValue	ComparedGenes
Npun_R2486	Npun_R5719	0.963800567	*murE-murA*
Npun_R1733	Npun_R1952	0.949753362	*LMW PBP-uppS*
Npun_F0446	Npun_F0447	0.945244621	*murC-murB*
Npun_F3659	Npun_F5597	0.911178829	***rpaA*** *-ddl*
Npun_F4452	Npun_R3281	0.876634722	Class B PBP-Class A PBP
Npun_R1302	Npun_R1733	0.866959791	***nagB*** - LMW PBP
Npun_F2411	Npun_R4507	0.858599638	*murG-bacA*
Npun_F0907	Npun_R5719	0.857098146	***glmU-*** *murA*
Npun_F0907	Npun_R4507	0.833793629	***glmU*** *-bacA*
Npun_F0907	Npun_R2557	0.833598268	***glmU*** -LMW PBP
Npun_F4453	Npun_R5719	0.829890155	Class B PBP-*murA*
Npun_F4453	Npun_R4507	0.822625392	Class B PBP-*bacA*
Npun_F0168	Npun_R3559	0.821610231	Class B PBP-*murT*
Npun_F0168	Npun_R2557	0.815816275	Class B PBP-LMW PBP
Npun_F0447	Npun_R3559	0.799008701	*murB-murT*
Npun_R2557	Npun_R5719	0.798527088	LMW PBP-*murA*
Npun_R2557	Npun_R4507	0.792949227	LMW PBP-*bacA*
Npun_F0907	Npun_F4453	0.786277386	***glmU*** -Class B PBP
Npun_F0446	Npun_R3559	0.779678986	*murC-murT*
Npun_F0168	Npun_F0907	0.774839495	Class B PBP-***glmU***
Npun_F2411	Npun_F4453	0.77106193	*murG-* Class B PBP
Npun_F2411	Npun_R5719	0.764573276	*murG-murA*
Npun_R1302	Npun_R1952	0.760076043	***nagB*** *-uppS*
Npun_F4453	Npun_R2486	0.758140407	Class B PBP-*murE*
Npun_F3925	Npun_F3940	0.750430543	*pgi-mraY*

Non-peptidoglycan Gene

Supplemental Table S2. Correlations of cell division, peptidoglycan biosynthesis, and sugar metabolism gene expressions.

Gene1	Gene2	CorrelationValue	ComparedGenes
Npun_R2486	Npun_R5719	0.963800567	MurE-MurA
Npun_F5138	Npun_R1839	0.954991591	FtsE-MreD
Npun_R1733	Npun_R1952	0.949753362	LMW PBP-UppS
Npun_F3647	Npun_F3659	0.946919803	MinC-RpaA
Npun_F0446	Npun_F0447	0.945244621	MurC-MurB
Npun_F5597	Npun_R4092	0.938576687	Ddl-FtsK
Npun_F4453	Npun_R4933	0.929808899	Class B PBP-Cdv1
Npun_F3647	Npun_R0056	0.921719228	MinC-DUF152
Npun_R1698	Npun_R2486	0.921037611	SepF-MurE
Npun_F3659	Npun_F5597	0.911178829	RpaA-Ddl
Npun_BF043	Npun_F0907	0.906259505	MinD-GlmU
Npun_F5138	Npun_R1733	0.900532737	FtsE-LMW PBP
Npun_F4452	Npun_R1841	0.898245014	Class B PBP-MreB
Npun_R2486	Npun_R4806	0.897717901	MurE-FtsQ
Npun_F4881	Npun_F5597	0.897189533	FtsH-Ddl
Npun_F3647	Npun_F5597	0.894821592	MinC-Ddl
Npun_R1698	Npun_R4806	0.89448759	SepF-FtsQ
Npun_F3659	Npun_R4092	0.891497607	RpaA-FtsK
Npun_F5214	Npun_R0056	0.889627672	GlmS-DUF152
Npun_R4806	Npun_R5719	0.889400819	FtsQ-MurA
Npun_F5138	Npun_R1952	0.886711221	FtsE-UppS
Npun_F3659	Npun_R6629	0.881969897	RpaA-SepI
Npun_R1698	Npun_R4804	0.880481335	SepF-FtsZ
Npun_R1840	Npun_R1841	0.879328505	MreC-MreB
Npun_R1698	Npun_R5719	0.878051425	SepF-MurA
Npun_F4452	Npun_R3281	0.876634722	Class B PBP-Class A PBP
Npun_F4881	Npun_R4092	0.875996454	FtsH-FtsK
Npun_R4933	Npun_R5149	0.875761242	Cdv1-GAF-HisKin
Npun_R4507	Npun_R5149	0.875678492	BacA-GAF-HisKin
Npun_F2411	Npun_R5149	0.875162279	MurG-GAF-HisKin
Npun_R6354	Npun_R6629	0.874779885	YlmH-SepI
Npun_R1733	Npun_R1839	0.870970857	LMW PBP-MreD
Npun_R1302	Npun_R1733	0.866959791	NagB-LMW PBP
Npun_R1839	Npun_R1841	0.866074827	MreD-MreB
Npun_R4507	Npun_R4933	0.863726036	BacA-Cdv1
Npun_F3659	Npun_R0056	0.863131187	RpaA-DUF152
Npun_BF043	Npun_R2557	0.860702956	MinD-LMW PBP
Npun_F3648	Npun_F3649	0.860169981	MinD-MinE
Npun_F3647	Npun_R6629	0.859454021	MinC-SepI
Npun_F2411	Npun_R4507	0.858599638	MurG-BacA
Npun_BF043	Npun_F0168	0.858532054	MinD-Class B PBP
Npun_R1839	Npun_R1840	0.857190145	MreD-MreC
Npun_F0907	Npun_R5719	0.857098146	GlmU-MurA
Npun_R1733	Npun_R1841	0.853753075	LMW PBP-MreB
Npun_F3647	Npun_R4092	0.845587208	MinC-FtsK
Npun_R1698	Npun_R1841	0.84248621	SepF-MreB
Npun_F2411	Npun_R4933	0.840462196	MurG-Cdv1
Npun_F3659	Npun_R6354	0.838489013	RpaA-YlmH
Npun_R1839	Npun_R1952	0.838013328	MreD-UppS
Npun_F5597	Npun_R0056	0.835556776	Ddl-DUF152
Npun_F0907	Npun_R4507	0.833793629	GlmU-BacA
Npun_F0907	Npun_R2557	0.833598268	GlmU-LMW PBP
Npun_F5597	Npun_R6354	0.832505749	Ddl-YlmH
Npun_F4453	Npun_R5719	0.829890155	Class B PBP-MurA
Npun_F0907	Npun_R5149	0.82564693	GlmU-GAF-HisKin
Npun_F4453	Npun_R4507	0.822625392	Class B PBP-BacA
Npun_F0168	Npun_R3559	0.821610231	Class B PBP-MurT
Npun_R4092	Npun_R6354	0.821325661	FtsK-YlmH
Npun_R1841	Npun_R1952	0.820090948	MreB-UppS
Npun_F0447	Npun_R4806	0.819999098	MurB-FtsQ
Npun_F0168	Npun_R2557	0.815816275	Class B PBP-LMW PBP
Npun_F5138	Npun_R1840	0.807280548	FtsE-MreC
Npun_F3659	Npun_F4881	0.805513371	RpaA-FtsH
Npun_F0907	Npun_R4806	0.805274401	GlmU-FtsQ
Npun_F5138	Npun_R1841	0.803919879	FtsE-MreB
Npun_F0447	Npun_R3559	0.799008701	MurB-MurT
Npun_R2557	Npun_R5719	0.798527088	LMW PBP-MurA
Npun_R2486	Npun_R4804	0.797299888	MurE-FtsZ
Npun_F0907	Npun_R4804	0.796054301	GlmU-FtsZ
Npun_R1698	Npun_R1840	0.79543787	SepF-MreC
Npun_R2557	Npun_R4507	0.792949227	LMW PBP-BacA
Npun_R4804	Npun_R5719	0.792599137	FtsZ-MurA
Npun_F3647	Npun_R6354	0.792508293	MinC-YlmH
Npun_F5597	Npun_R6629	0.789144362	Ddl-SepI
Npun_F4453	Npun_R4804	0.786955439	Class B PBP-FtsZ
Npun_R2557	Npun_R4804	0.786854897	LMW PBP-FtsZ
Npun_R1841	Npun_R3281	0.786346127	MreB-Class A PBP
Npun_F0907	Npun_F4453	0.786277386	GlmU-Class B PBP
Npun_BF043	Npun_R4806	0.785448443	MinD-FtsQ
Npun_R1952	Npun_R4806	0.782383771	UppS-FtsQ
Npun_R1841	Npun_R4806	0.78114049	MreB-FtsQ
Npun_R3910	Npun_R4092	0.779751668	BolA-FtsK
Npun_F0446	Npun_R3559	0.779678986	MurC-MurT
Npun_R4933	Npun_R5719	0.775928629	Cdv1-MurA
Npun_F0168	Npun_F0907	0.774839495	Class B PBP-GlmU
Npun_F4453	Npun_R5149	0.773071614	Class B PBP-GAF-HisKin
Npun_F0907	Npun_R4933	0.771274521	GlmU-Cdv1
Npun_F2411	Npun_F4453	0.77106193	MurG-Class B PBP
Npun_R4092	Npun_R6629	0.770623908	FtsK-SepI
Npun_R1698	Npun_R1839	0.767269352	SepF-MreD
Npun_BF043	Npun_R5719	0.765534647	MinD-MurA
Npun_F2411	Npun_R5719	0.764573276	MurG-MurA
Npun_F5138	Npun_R1302	0.762791289	FtsE-NagB
Npun_R1302	Npun_R1952	0.760076043	NagB-UppS
Npun_F3647	Npun_F5214	0.759735932	MinC-GlmS
Npun_F3647	Npun_F4881	0.758607248	MinC-FtsH
Npun_R4804	Npun_R4806	0.758602916	FtsZ-FtsQ
Npun_F4453	Npun_R2486	0.758140407	Class B PBP-MurE
Npun_BF043	Npun_R4009	0.75431246	MinD-MurD
Npun_F4452	Npun_R1839	0.752967292	Class B PBP-MreD
Npun_F4881	Npun_R0056	0.752668922	FtsH-DUF152
Npun_BF043	Npun_R4804	0.751019748	MinD-FtsZ
Npun_F3925	Npun_F3940	0.750430543	Pgi-MraY

Supplemental Table S3. BLASTp results of *Synechococcus elongatus* CikA query.

Locus Tag	Accession	Name	Bit-Score	E Value	% Pairwise Identity	% Identical Sites	Hit Start	Hit End	Query Start	Query End	% Query Coverage
Npun_R1685	ACC80356.1	CP001037 - multi-sensor hybrid histidine kinase	219.935	1.63E-60	32.00	32.00	2083	3672	159	707	72.81%
Npun_R3691	ACC82081.1	CP001037 - multi-sensor hybrid histidine kinase	231.876	1.91E-64	33.70	33.70	3559	5163	159	682	69.50%
Npun_F5035	ACC83378.1	CP001037 - multi-sensor hybrid histidine kinase	160.999	3.17E-41	28.20	28.20	3538	5109	159	677	68.83%
Npun_F2363	ACC80931.1	CP001037 - multi-sensor hybrid histidine kinase	186.037	2.58E-49	29.80	29.80	1414	2943	159	626	62.07%
Npun_F1185	ACC79910.1	CP001037 - multi-sensor signal transduction histidine kinase	181.03	2.37E-49	29.60	29.60	391	1716	158	621	61.54%
Npun_F6362	ACC84634.1	CP001037 - multi-sensor signal transduction histidine kinase	155.606	7.85E-40	27.20	27.20	1249	2547	149	611	61.41%
Npun_F1000	ACC79731.1	CP001037 - GAF sensor signal transduction histidine kinase	439.884	2.49E-145	50.50	50.50	589	2037	150	612	61.41%
Npun_R4776	ACC83125.1	CP001037 - multi-sensor signal transduction histidine kinase	108.997	6.00E-25	23.40	23.40	1852	3285	154	608	60.34%
Npun_R6149	ACC84435.1	CP001037 - multi-sensor signal transduction histidine kinase	133.265	1.13E-32	24.10	24.10	1210	2586	158	608	59.81%
Npun_R1550	ACC80239.1	CP001037 - GAF sensor signal transduction histidine kinase	159.844	6.36E-42	29.80	29.80	460	1698	158	608	59.81%
Npun_F1203	ACC79928.1	CP001037 - multi-sensor signal transduction histidine kinase	88.1965	1.51E-18	23.90	23.90	1282	2805	159	608	59.68%
Npun_R0896	ACC79633.1	CP001037 - multi-sensor hybrid histidine kinase	161.77	1.25E-41	28.80	28.80	1312	2703	263	710	59.42%
Npun_F5679	ACC83981.1	CP001037 - multi-sensor hybrid multi-kinase	145.206	4.37E-36	27.40	27.40	5743	7104	282	727	59.15%
Npun_R2903	ACC81436.1	CP001037 - multi-sensor signal transduction histidine kinase	154.836	2.04E-39	26.10	26.10	2089	3324	161	605	59.02%
Npun_R5149	ACC83481.1	CP001037 - GAF sensor signal transduction histidine kinase	153.295	1.66E-39	29.60	29.60	724	1959	165	608	58.89%
Npun_AF142	ACC85008.1	CP001038 - GAF sensor signal transduction histidine kinase	128.642	4.77E-31	26.90	26.90	2161	3543	167	608	58.62%
Npun_R5113	ACC83447.1	CP001037 - GAF sensor signal transduction histidine kinase	97.8265	1.83E-21	23.20	23.20	1696	3006	167	608	58.62%
Npun_R1597	ACC80282.1	CP001037 - GAF sensor signal transduction histidine kinase	118.627	6.15E-28	25.30	25.30	1738	3108	167	608	58.62%
Npun_F2854	ACC81387.1	CP001037 - GAF sensor signal transduction histidine kinase	129.413	2.66E-31	25.00	25.00	1741	3144	167	608	58.62%
Npun_R6125	ACC84413.1	CP001037 - multi-sensor signal transduction histidine kinase	99.3673	4.80E-22	26.20	26.20	1060	2247	169	608	58.36%
Npun_R5313	ACC83634.1	CP001037 - multi-sensor signal transduction histidine kinase	105.916	5.09E-24	26.00	26.00	1315	2697	170	608	58.22%
Npun_F2781	ACC81314.1	CP001037 - GAF sensor signal transduction histidine kinase	107.842	9.55E-25	28.50	28.50	643	1971	159	595	57.96%
Npun_F4131	ACC82509.1	CP001037 - GAF sensor hybrid histidine kinase	144.05	7.41E-37	27.50	27.50	328	1551	278	709	57.29%
Npun_R6464	ACC84732.1	CP001037 - multi-sensor hybrid histidine kinase	161.384	7.12E-42	31.10	31.10	967	2118	293	698	53.85%
Npun_F5092	ACC83426.1	CP001037 - multi-sensor hybrid histidine kinase	149.443	1.15E-37	27.90	27.90	2290	3585	291	694	53.58%
Npun_R3548	ACC81949.1	CP001037 - multi-sensor hybrid histidine kinase	132.494	3.19E-32	28.30	28.30	2914	4230	302	694	52.12%
Npun_R5897	ACC84193.1	CP001037 - multi-sensor hybrid histidine kinase	183.726	1.28E-48	30.70	30.70	3055	4251	295	677	50.80%
Npun_F1211	ACC79935.1	CP001037 - integral membrane sensor hybrid histidine kinase	174.481	6.76E-46	34.70	34.70	676	1797	321	697	50.00%
Npun_R3825	ACC82209.1	CP001037 - hybrid histidine kinase	211.846	4.84E-60	35.40	35.40	571	1698	331	706	49.87%
Npun_F1600	ACC80285.1	CP001037 - integral membrane sensor hybrid histidine kinase	162.54	1.13E-42	34.40	34.40	382	1491	351	705	47.08%
Npun_F5479	ACC83786.1	CP001037 - integral membrane sensor hybrid histidine kinase	185.267	1.97E-49	38.10	38.10	1300	2376	351	697	46.02%
Npun_F2686	ACC81226.1	CP001037 - GAF sensor hybrid histidine kinase	184.882	6.58E-49	34.90	34.90	4510	5658	360	704	45.76%
Npun_R2035	ACC80662.1	CP001037 - PAS/PAC sensor hybrid histidine kinase	142.51	1.91E-35	32.10	32.10	2143	3189	384	727	45.62%
Npun_R1432	ACC80139.1	CP001037 - GAF sensor signal transduction histidine kinase	78.9518	4.79E-16	27.30	27.30	277	1254	265	608	45.62%
Npun_R6347	ACC84619.1	CP001037 - multi-sensor hybrid histidine kinase	169.859	1.69E-44	36.50	36.50	949	1986	355	694	45.09%
Npun_R1798	ACC80460.1	CP001037 - multi-sensor hybrid histidine kinase	169.859	2.89E-44	35.80	35.80	1876	2913	384	719	44.56%
Npun_R4748	ACC83100.1	CP001037 - multi-sensor hybrid histidine kinase	239.58	3.86E-69	40.50	40.50	1084	2118	384	714	43.90%
Npun_R2262	ACC80851.1	CP001037 - PAS/PAC sensor hybrid histidine kinase	134.035	3.23E-33	31.30	31.30	844	1869	384	710	43.37%
Npun_F6350	ACC84622.1	CP001037 - PAS/PAC sensor hybrid histidine kinase	155.606	8.20E-40	34.40	34.40	1525	2535	384	710	43.37%
Npun_R2272	ACC80860.1	CP001037 - multi-sensor signal transduction multi-kinase	100.523	3.67E-22	26.60	26.60	4387	5403	289	615	43.37%
Npun_F2908	ACC81440.1	CP001037 - multi-sensor hybrid histidine kinase	160.614	3.89E-41	33.70	33.70	3127	4140	384	710	43.37%
Npun_R4028	ACC82407.1	CP001037 - GAF sensor signal transduction histidine kinase	86.2705	2.40E-18	25.50	25.50	328	1335	283	609	43.37%
Npun_R2263	ACC80852.1	CP001037 - hybrid histidine kinase	162.155	3.18E-42	34.00	34.00	649	1650	384	707	42.97%
Npun_R2375	ACC80942.1	CP001037 - multi-sensor hybrid histidine kinase	193.741	5.44E-52	34.20	34.20	1582	2697	384	704	42.57%
Npun_DR038	ACC85457.1	CP001041 - putative PAS/PAC sensor protein	158.688	1.33E-40	34.80	34.80	2782	3741	384	701	42.18%
Npun_F6040	ACC84330.1	CP001037 - multi-sensor signal transduction histidine kinase	176.022	1.95E-46	34.40	34.40	1732	2640	291	608	42.18%
Npun_F2346	ACC80914.1	CP001037 - multi-sensor hybrid histidine kinase	142.895	1.78E-35	33.00	33.00	1135	2016	311	626	41.91%
Npun_R1760	ACC80422.1	CP001037 - GAF sensor hybrid histidine kinase	170.629	1.35E-45	35.40	35.40	661	1608	384	698	41.78%
Npun_R2209	ACC80805.1	CP001037 - GAF sensor signal transduction histidine kinase	79.7221	2.46E-16	28.50	28.50	367	1269	296	608	41.51%
Npun_AR131	ACC85002.1	CP001038 - multi-sensor hybrid histidine kinase	159.073	5.62E-41	36.40	36.40	1126	2088	384	695	41.38%
Npun_R3083	ACC81570.1	CP001037 - multi-sensor hybrid histidine kinase	138.658	1.51E-34	30.80	30.80	874	1929	384	695	41.38%
Npun_R2268	ACC80857.1	CP001037 - PAS/PAC sensor hybrid histidine kinase	146.362	4.36E-37	33.10	33.10	1033	1968	384	694	41.25%
Npun_R1868	ACC80525.1	CP001037 - multi-sensor hybrid histidine kinase	133.65	5.13E-33	30.50	30.50	1018	1968	384	694	41.25%
Npun_F2346	ACC80914.1	CP001037 - multi-sensor hybrid histidine kinase	143.28	1.24E-35	31.00	31.00	2611	3555	384	694	41.25%
Npun_R3572	ACC81968.1	CP001037 - multi-sensor hybrid histidine kinase	151.369	3.28E-38	35.50	35.50	3388	4320	384	694	41.25%
Npun_R3591	ACC81987.1	CP001037 - multi-sensor hybrid histidine kinase	164.081	2.21E-42	33.20	33.20	1987	2949	384	694	41.25%
Npun_F5360	ACC83673.1	CP001037 - multi-sensor hybrid histidine kinase	165.236	1.21E-42	36.10	36.10	2044	2973	384	682	39.66%
Npun_R4744	ACC83096.1	CP001037 - PAS/PAC sensor hybrid histidine kinase	150.984	3.62E-38	33.80	33.80	1948	2913	384	677	38.99%
Npun_F2889	ACC81422.1	CP001037 - CBS sensor hybrid histidine kinase	194.512	3.13E-52	36.10	36.10	1165	2172	384	677	38.99%
Npun_R1449	ACC80152.1	CP001037 - response regulator receiver sensor signal transduction histidine kinase	183.726	1.80E-51	38.80	38.80	337	1224	333	620	38.20%
Npun_R1759	ACC80421.1	CP001037 - GAF sensor signal transduction histidine kinase	160.999	7.33E-42	39.00	39.00	1219	2058	322	608	38.06%
Npun_R3054	ACC81549.1	CP001037 - Chase sensor signal transduction histidine kinase	118.242	7.15E-29	32.50	32.50	466	1284	327	610	37.67%
Npun_F0022	ACC78825.1	CP001037 - response regulator receiver sensor signal transduction histidine kinase	128.642	8.95E-33	33.20	33.20	352	1149	335	608	36.34%
Npun_F1439	ACC80146.1	CP001037 - integral membrane sensor signal transduction histidine kinase	150.214	1.87E-39	34.00	34.00	631	1413	342	608	35.41%
Npun_R1236	ACC79960.1	CP001037 - integral membrane sensor signal transduction histidine kinase	143.28	4.92E-36	34.40	34.40	1033	1830	344	608	35.15%
Npun_R1012	ACC79743.1	CP001037 - integral membrane sensor signal transduction histidine kinase HepK	177.178	4.82E-48	42.70	42.70	910	1707	350	608	34.35%
Npun_R3198	ACC81651.1	CP001037 - PAS/PAC sensor signal transduction histidine kinase	109.383	2.59E-26	29.10	29.10	409	1137	358	615	34.22%
Npun_R4211	ACC82586.1	CP001037 - PAS/PAC sensor hybrid histidine kinase	142.895	1.28E-35	37.10	37.10	2194	2982	384	641	34.22%
Npun_F6002	ACC84292.1	CP001037 - multi-sensor signal transduction histidine kinase	110.153	6.60E-26	32.70	32.70	763	1482	355	608	33.69%
Npun_F0020	ACC78823.1	CP001037 - multi-sensor signal transduction histidine kinase	96.6709	3.37E-21	30.70	30.70	1504	2235	357	608	33.42%
Npun_F3565	ACC81961.1	CP001037 - multi-sensor signal transduction multi-kinase	103.219	5.58E-23	29.90	29.90	5428	6129	356	603	32.89%
Npun_F3675	ACC82065.1	CP001037 - multi-sensor signal transduction histidine kinase	88.9669	3.73E-19	30.00	30.00	787	1479	359	603	32.49%
Npun_BF140	ACC85272.1	CP001039 - integral membrane sensor signal transduction histidine kinase	99.3673	8.39E-23	30.00	30.00	574	1239	386	612	30.11%
Npun_R0454	ACC79233.1	CP001037 - GAF sensor signal transduction histidine kinase	95.5153	3.49E-21	28.40	28.40	688	1518	384	610	30.11%
Npun_F0839	ACC79577.1	CP001037 - response regulator receiver sensor signal transduction histidine kinase	116.701	1.07E-28	31.30	31.30	430	1137	386	611	29.97%
Npun_R1448	ACC80151.1	CP001037 - response regulator receiver sensor signal transduction histidine kinase	117.857	3.33E-29	29.40	29.40	430	1119	386	610	29.84%
Npun_F0957	ACC79688.1	CP001037 - response regulator receiver sensor signal transduction histidine kinase	121.324	2.29E-30	32.00	32.00	424	1077	384	608	29.84%
Npun_F4953	ACC83298.1	CP001037 - PAS/PAC sensor signal transduction histidine kinase	129.028	2.13E-32	35.60	35.60	667	1326	384	608	29.84%
Npun_F3541	ACC81945.1	CP001037 - PAS/PAC sensor hybrid histidine kinase	134.42	2.75E-33	40.40	40.40	880	1569	384	608	29.84%
Npun_F3797	ACC82181.1	CP001037 - multi-sensor signal transduction histidine kinase	106.686	2.10E-24	32.30	32.30	1582	2244	384	608	29.84%
Npun_F5043	ACC83382.1	CP001037 - multi-sensor signal transduction histidine kinase	150.984	1.80E-39	36.30	36.30	817	1521	384	608	29.84%
Npun_F2908	ACC81440.1	CP001037 - multi-sensor hybrid histidine kinase	135.191	4.66E-33	34.50	34.50	1054	1719	384	608	29.84%
Npun_R4769	ACC83120.1	CP001037 - multi-component transcriptional regulator, winged helix family	166.007	4.60E-43	41.30	41.30	2152	2814	384	608	29.84%
Npun_F1330	ACC80042.1	CP001037 - histidine kinase	132.88	3.86E-34	37.70	37.70	496	1179	386	610	29.84%
Npun_R6203	ACC84489.1	CP001037 - integral membrane sensor signal transduction histidine kinase	92.4337	1.68E-20	30.90	30.90	571	1224	386	608	29.58%
Npun_F5193	ACC83525.1	CP001037 - integral membrane sensor signal transduction histidine kinase	96.6709	1.31E-21	29.80	29.80	694	1467	386	608	29.58%
Npun_F0303	ACC79088.1	CP001037 - integral membrane sensor signal transduction histidine kinase	100.138	2.95E-23	29.30	29.30	412	1080	386	608	29.58%
Npun_R6227	ACC84511.1	CP001037 - integral membrane sensor signal transduction histidine kinase	123.25	4.81E-31	38.20	38.20	433	1080	386	608	29.58%
Npun_F1277	ACC79998.1	CP001037 - PAS/PAC sensor signal transduction histidine kinase	114.39	6.60E-27	35.00	35.00	1288	1935	384	605	29.44%
Npun_R3052	ACC81547.1	CP001037 - integral membrane sensor signal transduction histidine kinase	135.961	1.40E-34	35.40	35.40	766	1410	387	608	29.44%
Npun_R3716	ACC82106.1	CP001037 - multi-sensor signal transduction histidine kinase	112.079	3.83E-26	29.80	29.80	1189	1860	384	603	29.18%
Npun_R2485	ACC81046.1	CP001037 - histidine kinase	118.627	1.40E-28	35.90	35.90	892	1539	386	605	29.18%
Npun_F3797	ACC82181.1	CP001037 - multi-sensor signal transduction histidine kinase	97.8265	1.38E-21	31.00	31.00	94	744	152	370	29.05%
Npun_R5113	ACC83447.1	CP001037 - GAF sensor signal transduction histidine kinase	82.0333	1.46E-16	30.60	30.60	1123	1713	163	371	27.72%
Npun_F1553	ACC80242.1	CP001037 - diguanylate cyclase with PAS/PAC and GAF sensors	89.3521	6.62E-19	30.10	30.10	1573	2142	152	360	27.72%
Npun_R1060	ACC79786.1	CP001037 - diguanylate cyclase/phosphodiesterase with GAF sensor	105.145	7.49E-24	34.50	34.50	1135	1689	158	360	26.92%
Npun_R6125	ACC84413.1	CP001037 - multi-sensor signal transduction histidine kinase	86.2705	6.36E-18	29.10	29.10	463	993	159	354	25.99%
Npun_R1597	ACC80282.1	CP001037 - GAF sensor signal transduction histidine kinase	82.0333	1.43E-16	31.50	31.50	85	621	165	359	25.86%
Npun_R3784	ACC82169.1	CP001037 - multi-sensor hybrid histidine kinase	93.5893	3.83E-20	32.30	32.30	76	591	162	355	25.73%
Npun_R3572	ACC81968.1	CP001037 - multi-sensor hybrid histidine kinase	90.1225	4.73E-19	32.80	32.80	421	981	167	353	24.80%

Supplemental Table S4. tBLASTn results for cyanobacterial CikA-like proteins.

Locus Tag	Accession	Name	Bit-Score	E Value	% Pairwise Identity	% Identical Sites	Hit Start	Hit End	Query Start	Query End	% Query Coverage
Npun_F1000	ACC79731.1	CP001037 - GAF sensor signal transduction histidine kinase	800.045	0.00E+00	70.50	70.30	1	2040	95	697	71.11
Npun_F2363	ACC80931.1	CP001037 - multi-sensor hybrid histidine kinase	159.458	1.66E-40	29.80	29.40	1411	3321	247	830	68.87
Npun_R1685	ACC80356.1	CP001037 - multi-sensor hybrid histidine kinase	222.631	8.97E-61	32.80	32.50	2083	3792	248	830	68.75
Npun_R3691	ACC82081.1	CP001037 - multi-sensor hybrid histidine kinase	230.72	2.03E-63	33.40	33.10	3556	5364	247	828	68.63
Npun_F2854	ACC81387.1	CP001037 - GAF sensor signal transduction histidine kinase	116.316	4.77E-27	26.50	26.40	1681	3144	236	692	53.89
Npun_R5897	ACC84193.1	CP001037 - multi-sensor hybrid histidine kinase	206.068	2.45E-55	31.40	31.30	3055	4458	379	829	53.18
Npun_F6362	ACC84634.1	CP001037 - multi-sensor signal transduction histidine kinase	146.362	1.10E-36	28.00	27.80	1276	2544	247	694	52.83
Npun_R6149	ACC84435.1	CP001037 - multi-sensor signal transduction histidine kinase	92.0485	1.39E-19	25.00	24.70	1210	2586	247	692	52.59
Npun_R5149	ACC83481.1	CP001037 - GAF sensor signal transduction histidine kinase	107.842	1.04E-24	26.90	26.60	712	1959	250	692	52.24
Npun_R4776	ACC83125.1	CP001037 - multi-sensor signal transduction histidine kinase	102.834	6.86E-23	25.80	25.60	1882	3285	251	692	52.12
Npun_R2903	ACC81436.1	CP001037 - multi-sensor signal transduction histidine kinase	160.614	6.82E-41	29.90	29.70	2089	3330	250	691	52.12
Npun_R5113	ACC83447.1	CP001037 - GAF sensor signal transduction histidine kinase	95.5153	1.09E-20	26.10	25.90	1684	3009	252	693	52.12
Npun_R1597	ACC80282.1	CP001037 - GAF sensor signal transduction histidine kinase	110.153	3.69E-25	28.00	27.80	1726	3108	252	692	52.00
Npun_F5479	ACC83786.1	CP001037 - integral membrane sensor hybrid histidine kinase	197.208	6.04E-53	38.30	38.10	1333	2496	446	820	44.22
Npun_F5679	ACC83981.1	CP001037 - multi-sensor hybrid multi-kinase	162.54	2.87E-41	33.20	33.10	5992	7107	468	830	42.81
Npun_R1868	ACC80525.1	CP001037 - multi-sensor hybrid histidine kinase	173.326	1.13E-45	33.20	33.00	1018	2133	468	830	42.81
Npun_R6464	ACC84732.1	CP001037 - multi-sensor hybrid histidine kinase	172.17	4.05E-45	34.60	34.40	1165	2271	468	830	42.81
Npun_F5092	ACC83426.1	CP001037 - multi-sensor hybrid histidine kinase	157.532	5.95E-40	30.50	30.30	2512	3750	468	830	42.81
Npun_F2889	ACC81422.1	CP001037 - CBS sensor hybrid histidine kinase	202.986	1.41E-54	32.80	32.40	1165	2373	468	830	42.81
Npun_R4211	ACC82586.1	CP001037 - PAS/PAC sensor hybrid histidine kinase	178.333	1.44E-46	34.50	34.30	2194	3309	468	829	42.69
Npun_R2035	ACC80662.1	CP001037 - PAS/PAC sensor hybrid histidine kinase	169.088	1.03E-43	34.30	34.10	2143	3237	468	829	42.69
Npun_R1798	ACC80460.1	CP001037 - multi-sensor hybrid histidine kinase	183.726	2.31E-48	36.50	36.30	1876	2994	468	829	42.69
Npun_R6347	ACC84619.1	CP001037 - multi-sensor hybrid histidine kinase	169.474	3.96E-44	34.20	34.00	1018	2142	468	829	42.69
Npun_R0896	ACC79633.1	CP001037 - multi-sensor hybrid histidine kinase	162.155	2.21E-41	30.50	30.30	1618	2811	468	829	42.69
Npun_F2908	ACC81440.1	CP001037 - multi-sensor hybrid histidine kinase	156.762	1.34E-39	31.60	31.50	3127	4239	468	829	42.69
Npun_R3548	ACC81949.1	CP001037 - multi-sensor hybrid histidine kinase	143.665	1.94E-35	31.30	31.10	3103	4392	468	829	42.69
Npun_R2263	ACC80852.1	CP001037 - hybrid histidine kinase	167.162	1.44E-43	33.20	32.70	649	1758	468	829	42.69
Npun_DR038	ACC85457.1	CP001041 - putative PAS/PAC sensor protein	179.489	6.34E-47	34.00	33.90	2782	3885	468	828	42.57
Npun_R2268	ACC80857.1	CP001037 - PAS/PAC sensor hybrid histidine kinase	190.274	2.01E-51	35.80	35.60	1033	2127	468	828	42.57
Npun_F6350	ACC84622.1	CP001037 - PAS/PAC sensor hybrid histidine kinase	173.326	3.32E-45	33.30	33.20	1525	2631	468	828	42.57
Npun_R4744	ACC83096.1	CP001037 - PAS/PAC sensor hybrid histidine kinase	170.244	6.10E-44	33.10	32.80	1948	3108	468	828	42.57
Npun_F3541	ACC81945.1	CP001037 - PAS/PAC sensor hybrid histidine kinase	167.162	9.06E-44	34.60	34.50	880	2025	468	828	42.57
Npun_F2908	ACC81440.1	CP001037 - multi-sensor hybrid histidine kinase	160.999	5.19E-41	29.80	29.60	1054	2277	468	828	42.57
Npun_F1600	ACC80285.1	CP001037 - integral membrane sensor hybrid histidine kinase	186.037	1.96E-50	37.30	37.20	490	1611	468	828	42.57
Npun_F1211	ACC79935.1	CP001037 - integral membrane sensor hybrid histidine kinase	181.415	8.90E-48	34.90	34.70	814	1941	468	828	42.57
Npun_R1760	ACC80422.1	CP001037 - GAF sensor hybrid histidine kinase	167.548	2.57E-44	34.80	34.70	661	1782	468	828	42.57
Npun_R3591	ACC81987.1	CP001037 - multi-sensor hybrid histidine kinase	196.438	1.64E-52	34.90	34.70	1987	3105	468	827	42.45
Npun_R3572	ACC81968.1	CP001037 - multi-sensor hybrid histidine kinase	182.956	7.12E-48	36.30	36.10	3388	4476	468	827	42.45
Npun_F2346	ACC80914.1	CP001037 - multi-sensor hybrid histidine kinase	162.54	1.75E-41	32.00	31.80	2611	3711	468	827	42.45
Npun_R3083	ACC81570.1	CP001037 - multi-sensor hybrid histidine kinase	154.451	1.46E-39	31.60	31.40	874	2082	468	827	42.45
Npun_F4131	ACC82509.1	CP001037 - GAF sensor hybrid histidine kinase	149.058	2.46E-38	31.40	31.20	559	1650	468	825	42.22
Npun_R2262	ACC80851.1	CP001037 - PAS/PAC sensor hybrid histidine kinase	160.229	1.50E-41	33.30	33.20	844	1953	468	824	42.10
Npun_F5035	ACC83378.1	CP001037 - multi-sensor hybrid histidine kinase	171.785	2.60E-44	32.70	32.50	4108	5313	468	824	42.10
Npun_R3825	ACC82209.1	CP001037 - hybrid histidine kinase	204.912	4.26E-57	37.80	37.60	697	1803	468	824	42.10
Npun_R2375	ACC80942.1	CP001037 - multi-sensor hybrid histidine kinase	205.297	3.41E-55	34.80	34.50	1582	2793	468	822	41.86
Npun_R3784	ACC82169.1	CP001037 - multi-sensor hybrid histidine kinase	95.1301	1.31E-20	27.80	27.60	1546	2628	475	829	41.86
Npun_F2686	ACC81226.1	CP001037 - GAF sensor hybrid histidine kinase	199.134	5.26E-53	34.60	34.40	4585	5748	468	820	41.63
Npun_AR131	ACC85002.1	CP001038 - multi-sensor hybrid histidine kinase	155.992	1.12E-39	34.60	34.40	1126	2202	468	816	41.16
Npun_R4748	ACC83100.1	CP001037 - multi-sensor hybrid histidine kinase	241.506	3.26E-69	42.60	42.40	1084	2175	468	816	41.16
Npun_F1185	ACC79910.1	CP001037 - multi-sensor signal transduction histidine kinase	137.117	2.45E-34	34.00	33.90	739	1677	364	692	38.80
Npun_F5360	ACC83673.1	CP001037 - multi-sensor hybrid histidine kinase	178.718	1.30E-46	39.20	39.00	2044	2991	468	771	35.85
Npun_F6040	ACC84330.1	CP001037 - multi-sensor signal transduction histidine kinase	172.17	6.97E-45	38.50	38.40	1792	2640	395	692	35.14
Npun_R1012	ACC79743.1	CP001037 - integral membrane sensor signal transduction histidine kinase HepK	161.384	2.36E-42	44.20	44.20	946	1707	446	692	29.13
Npun_R1550	ACC80239.1	CP001037 - GAF sensor signal transduction histidine kinase	135.961	8.06E-34	36.00	36.00	994	1716	468	696	27.00
Npun_F0303	ACC79088.1	CP001037 - integral membrane sensor signal transduction histidine kinase	120.553	4.31E-30	32.60	32.60	406	1089	468	695	26.89
Npun_R5764	ACC84062.1	CP001037 - histidine kinase with KaiB domain, SasA	93.9745	5.35E-21	30.10	30.10	505	1200	468	695	26.89
Npun_BF140	ACC85272.1	CP001039 - integral membrane sensor signal transduction histidine kinase	103.219	7.15E-24	31.40	31.30	574	1239	470	696	26.77
Npun_R1449	ACC80152.1	CP001037 - response regulator receiver sensor signal transduction histidine kinase	184.496	2.72E-51	44.00	44.00	520	1194	468	694	26.77
Npun_R1448	ACC80151.1	CP001037 - response regulator receiver sensor signal transduction histidine kinase	119.398	1.21E-29	30.00	30.00	424	1119	468	694	26.77
Npun_R0454	ACC79233.1	CP001037 - GAF sensor signal transduction histidine kinase	98.9821	3.30E-22	30.50	30.50	688	1518	468	694	26.77
Npun_R3054	ACC81549.1	CP001037 - Chase sensor signal transduction histidine kinase	118.627	6.27E-29	34.40	34.40	619	1284	468	694	26.77
Npun_F0957	ACC79688.1	CP001037 - response regulator receiver sensor signal transduction histidine kinase	119.783	8.57E-30	32.00	32.00	424	1077	468	692	26.53
Npun_F0022	ACC78825.1	CP001037 - response regulator receiver sensor signal transduction histidine kinase	116.316	1.89E-28	31.90	31.90	487	1149	468	692	26.53
Npun_F0839	ACC79577.1	CP001037 - response regulator receiver sensor signal transduction histidine kinase	113.62	1.36E-27	31.00	31.00	424	1128	468	692	26.53
Npun_F4953	ACC83298.1	CP001037 - PAS/PAC sensor signal transduction histidine kinase	129.028	2.86E-32	34.70	34.70	667	1326	468	692	26.53
Npun_F1277	ACC79998.1	CP001037 - PAS/PAC sensor signal transduction histidine kinase	120.168	1.34E-28	35.40	35.30	1288	1944	468	692	26.53
Npun_R3198	ACC81651.1	CP001037 - PAS/PAC sensor signal transduction histidine kinase	113.62	1.29E-27	34.50	34.50	457	1116	468	692	26.53
Npun_F5043	ACC83382.1	CP001037 - multi-sensor signal transduction histidine kinase	149.443	8.80E-39	36.40	36.40	817	1521	468	692	26.53
Npun_F6002	ACC84292.1	CP001037 - multi-sensor signal transduction histidine kinase	122.479	7.89E-30	33.70	33.60	817	1482	468	692	26.53
Npun_F3797	ACC82181.1	CP001037 - multi-sensor signal transduction histidine kinase	118.627	5.01E-28	35.00	34.90	1582	2244	468	692	26.53
Npun_R6125	ACC84413.1	CP001037 - multi-sensor signal transduction histidine kinase	85.1149	1.50E-17	29.30	29.30	1573	2247	468	692	26.53
Npun_F2346	ACC80914.1	CP001037 - multi-sensor hybrid histidine kinase	137.502	1.22E-33	39.40	39.40	1297	1962	468	692	26.53
Npun_R4769	ACC83120.1	CP001037 - multi-component transcriptional regulator, winged helix family	158.688	2.31E-40	41.70	41.70	2152	2814	468	692	26.53
Npun_R6227	ACC84511.1	CP001037 - integral membrane sensor signal transduction histidine kinase	132.109	5.44E-34	39.70	39.60	427	1080	468	692	26.53
Npun_R1236	ACC79960.1	CP001037 - integral membrane sensor signal transduction histidine kinase	135.961	1.61E-33	37.70	37.70	1153	1830	468	692	26.53
Npun_F1330	ACC80042.1	CP001037 - histidine kinase	133.265	3.99E-34	36.20	36.20	496	1179	470	694	26.53
Npun_R1759	ACC80421.1	CP001037 - GAF sensor signal transduction histidine kinase	133.65	8.52E-33	40.70	40.60	1393	2058	468	692	26.53
Npun_F3565	ACC81961.1	CP001037 - multi-sensor signal transduction multi-kinase	92.4337	1.47E-19	29.00	28.90	5500	6150	471	694	26.42
Npun_R2485	ACC81046.1	CP001037 - histidine kinase	101.679	5.37E-23	33.10	33.00	886	1545	468	691	26.42
Npun_F0020	ACC78823.1	CP001037 - multi-sensor signal transduction histidine kinase	87.4261	3.34E-18	31.20	31.10	1591	2235	470	692	26.30
Npun_F1439	ACC80146.1	CP001037 - integral membrane sensor signal transduction histidine kinase	133.265	1.38E-33	35.90	35.90	763	1413	470	692	26.30
Npun_F5193	ACC83525.1	CP001037 - integral membrane sensor signal transduction histidine kinase	111.694	2.48E-26	32.50	32.40	694	1467	470	692	26.30
Npun_R6203	ACC84489.1	CP001037 - integral membrane sensor signal transduction histidine kinase	100.523	4.58E-23	30.50	30.50	571	1224	470	692	26.30
Npun_R3052	ACC81547.1	CP001037 - integral membrane sensor signal transduction histidine kinase	133.265	1.33E-33	36.10	36.00	766	1410	471	692	26.18
Npun_R3716	ACC82106.1	CP001037 - multi-sensor signal transduction histidine kinase	121.324	4.61E-29	32.80	32.80	1189	1860	468	687	25.94
Npun_F3675	ACC82065.1	CP001037 - multi-sensor signal transduction histidine kinase	93.2041	2.55E-20	29.30	29.20	850	1479	471	687	25.59
Npun_R6125	ACC84413.1	CP001037 - multi-sensor signal transduction histidine kinase	89.7373	5.26E-19	32.40	32.10	433	939	238	420	21.58
Npun_F1553	ACC80242.1	CP001037 - diguanylate cyclase with PAS/PAC and GAF sensors	97.8265	2.23E-21	36.90	36.80	1573	2070	241	420	21.23
Npun_R3784	ACC82169.1	CP001037 - multi-sensor hybrid histidine kinase	111.694	1.11E-25	39.90	39.50	43	534	244	420	20.87
Npun_F3797	ACC82181.1	CP001037 - multi-sensor signal transduction histidine kinase	89.3521	8.02E-19	33.40	33.30	106	642	245	420	20.75
Npun_R1597	ACC80282.1	CP001037 - GAF sensor signal transduction histidine kinase	93.5893	5.20E-20	33.60	33.50	67	558	247	422	20.75
Npun_R5113	ACC83447.1	CP001037 - GAF sensor signal transduction histidine kinase	97.8265	2.45E-21	35.00	34.90	55	537	251	422	20.28
Npun_F1883	ACC80538.1	CP001037 - putative GAF sensor protein	95.5153	1.07E-20	37.90	37.50	1504	1986	251	420	20.05
Npun_F1883	ACC80538.1	CP001037 - putative GAF sensor protein	89.7373	6.90E-19	35.80	35.40	2020	2502	251	420	20.05
Npun_R4776	ACC83125.1	CP001037 - multi-sensor signal transduction histidine kinase	90.1225	6.14E-19	32.90	32.60	1327	1815	251	420	20.05
Npun_R3572	ACC81968.1	CP001037 - multi-sensor hybrid histidine kinase	90.8929	3.98E-19	31.30	31.20	406	930	251	420	20.05
Npun_R1060	ACC79786.1	CP001037 - diguanylate cyclase/phosphodiesterase with GAF sensor	127.102	1.25E-30	43.10	42.70	1147	1617	251	420	20.05
Npun_R5113	ACC83447.1	CP001037 - GAF sensor signal transduction histidine kinase	85.5001	1.61E-17	33.10	32.90	1129	1599	254	421	19.81
Npun_AF142	ACC85008.1	CP001038 - GAF sensor signal transduction histidine kinase	94.7449	1.98E-20	35.30	35.20	2161	2625	256	420	19.46
Npun_R5313	ACC83634.1	CP001037 - multi-sensor signal transduction histidine kinase	88.1965	1.82E-18	33.20	33.10	1315	1755	259	421	19.22

Supplemental Table S5. Putative CikA homologue expression correlation.

Gene	CikA-like_Gene	CorrelationValue
Npun_R1733 (LMW PBP)	Npun_F2854	0.982449443
Npun_R1733 (LMW PBP)	Npun_R5113	0.971446745
Npun_R1952 (UppS)	Npun_R1685	0.968856927
Npun_R1733 (LMW PBP)	Npun_R1685	0.968649658
Npun_R1733 (LMW PBP)	Npun_F2363	0.954675064
Npun_R1733 (LMW PBP)	Npun_R1597	0.949939303
Npun_F5138 (FtsE)	Npun_R5113	0.94517937
Npun_F5138 (FtsE)	Npun_F2363	0.938532519
Npun_R1839 (MreD)	Npun_R5113	0.927348683
Npun_R1952 (UppS)	Npun_F2363	0.921459773
Npun_R1952 (UppS)	Npun_R5113	0.916957407
Npun_R0056 (DUF152)	Npun_R6149	0.916119909
Npun_R4092 (FtsK)	Npun_F6362	0.914128122
Npun_F5138 (FtsE)	Npun_R1597	0.911793486
Npun_R1841 (MreB)	Npun_R5113	0.911662811
Npun_R1839 (MreD)	Npun_R1597	0.908563261
Npun_R1952 (UppS)	Npun_F2854	0.905274232
Npun_F3659 (RpaA)	Npun_R3691	0.899298892
Npun_F5138 (FtsE)	Npun_F2854	0.892876802
Npun_R1841 (MreB)	Npun_F2854	0.886233362
Npun_F4881 (FtsH)	Npun_F6362	0.885568809
Npun_R1839 (MreD)	Npun_F2854	0.885381621
Npun_F5138 (FtsE)	Npun_R1685	0.881389818
Npun_F3647 (MinC)	Npun_R3691	0.880990067
Npun_R1302 (NagB)	Npun_R1597	0.87842755
Npun_R4933 (Cdv1)	Npun_R5149	0.875761242
Npun_R4507 (BacA)	Npun_R5149	0.875678492
Npun_F5214 (GlmS)	Npun_R6149	0.875275584
Npun_F2411 (MurG)	Npun_R5149	0.875162279
Npun_R1302 (NagB)	Npun_F2363	0.873216278
Npun_R1840 (MreC)	Npun_R1597	0.870303677
Npun_R1839 (MreD)	Npun_F2363	0.868200199
Npun_R1840 (MreC)	Npun_R5113	0.864124214
Npun_R3910 (BolA)	Npun_F6362	0.855022767
Npun_R1302 (NagB)	Npun_F2854	0.854136988
Npun_R1841 (MreB)	Npun_R1597	0.84607166
Npun_F3659 (RpaA)	Npun_F6362	0.840567752
Npun_R1302 (NagB)	Npun_R4776	0.837799452
Npun_R1952 (UppS)	Npun_R1597	0.837440445

Supplemental Table S6. Loci associated with motility and/or hormogonia development.

Locus Tag	Gene Name	Function	Mutant Phenotype
Npun_F0676	pilA	major pilin	non-motile
Npun_R0116	pilC	innermembrane platform	non-motile
Npun_R0117	pilT1	pilus retraction ATPase	non-motile, hyperpiliated
Npun_R0118	pilB	pilus extension ATPase	non-motile, loss of pilus extension
Npun_F2507	pilT2	pilus retraction ATPase	reduced motility
Npun_F5005	pilM	pilus alignment complex protein	non-motile
Npun_F5006	pilN	pilus alignment complex protein	
Npun_F5007	pilO	pilus alignment complex protein	
Npun_F5008	pilQ	outermembrane secretin	non-motile
Npun_F5230	hfq	Hfq homolog, essential for PilB activity	non-motile, loss of pilus extension
Npun_F4125	ebsA	polysaccharide biosynthesis	non-motile
Npun_F0677	ogtA	O-linked-B-N-acetylglucosamine transferase	non-motile, fails to accumulate PilA
Npun_F0066	hpsA	conserved hypothetical membrane protein	non-motile, reduced HPS
Npun_F0067	hpsB	minor pilin	non-motile, reduced HPS
Npun_F0068	hpsC	minor pilin	non-motile, reduced HPS
Npun_F0069	hpsD	minor pilin	non-motile, reduced HPS
Npun_F0070	hpsE	glycosyl transferase	non-motile, HPS-
Npun_F0071	hpsF	glycosyl transferase	non-motile, HPS-
Npun_F0072	hpsG	glycosyl transferase	non-motile, HPS-
Npun_F0073	hpsH	minor pilin	
Npun_F0075	hpsI	glycosyl transferase	
Npun_F0077	hpsJ	conserved hypothetical membrane protein	reduced motility, altered HPS composition
Npun_F0078	hpsK	glycosyl transferase	
Npun_R0640	hpsL	O-antigen ligase-like membrane protein	non-motile, HPS-
Npun_R0639	hpsM	glycosyl transferase	reduced motiltiy
Npun_R0638	hpsN	glycosyl transferase	non-motile, HPS-
Npun_R0637	hpsO	glycosyl transferase	non-motile, HPS-
Npun_R0636	hpsP	glycosyl transferase	non-motile, HPS-
Npun_F1388	hpsQ	glycosyl transferase	non-motile, HPS-
Npun_R1506	hpsR	glycosyl transferase	non-motile, HPS-
Npun_R5614	hpsS	glycosyl transferase	non-motile, HPS-
Npun_R5613	hpsT	glycosyl transferase	
Npun_R6512	hpsU	WcaF	
Npun_R0453	wzy	polysaccharide polymerase	
Npun_F0458	wza	polysaccharide export outer membrane protein	
Npun_F0459	wzc	polysaccharide co-polymerase	
Npun_F5960	hmpA	PatA-type response regulator	motile
Npun_F5961	hmpB	CheY-type response regulator	non-motile, reduced HPS
Npun_F5962	hmpC	CheW	non-motile, reduced HPS
Npun_F5963	hmpD	MCP	non-motile, reduced HPS
Npun_F5964	hmpE	CheA	non-motile, reduced HPS
Npun_R5959	hmpF	coiled-coil protein	non-motile, loss of pilus extension
Npun_F2161	ptxA	CheY, PatA family	
Npun_F2162	ptxB	CheY	
Npun_F2163	ptxC	CheW	
Npun_F2164	ptxD	MCP	motile, loss of phototaxis
Npun_F2165	ptxE	CheA	motile, loss of phototaxis

Supplemental Table S7. Expression correlation matrix for DGR-associated genes.

	Npun_F4884	Npun_F4885	Npun_F4886	Npun_F4887	Npun_F4888	**Npun_F4889**	**Npun_F4890**	**Npun_F4891**	**Npun_F4892**	**DGR-TR**	Npun_F4893	Npun_F4894	Npun_F4895	Npun_F4896	Npun_F4897	Annotation
Npun_F4884	1.0000	0.1827	0.3200	0.5340	0.3097	-0.2250	0.0000	0.7209	0.0637	-0.4366	0.3529	0.2061	-0.3143	0.0880	-0.2436	Hypothetical protein
Npun_F4885	0.1827	1.0000	0.8855	0.8370	0.6940	-0.1011	0.7616	0.2538	0.1663	0.2213	0.3876	-0.0107	0.0355	0.3152	-0.1244	CHAT domain protein
Npun_F4886	0.3200	0.8855	1.0000	0.8116	0.9064	0.2296	0.7382	0.3127	0.4173	-0.0526	0.5285	-0.2514	0.0305	0.6137	0.0696	Hypothetical protein
Npun_F4887	0.5340	0.8370	0.8116	1.0000	0.7205	-0.0777	0.5562	0.3386	0.1605	0.1880	0.4294	-0.0774	0.0242	0.4239	-0.1876	MoxR ATPase
Npun_F4888	0.3097	0.6940	0.9064	0.7205	1.0000	0.4065	0.7399	0.3002	0.6518	-0.0192	0.6820	-0.3750	0.0369	0.7537	0.3606	VWA protein
**Npun_F4889**	-0.2250	-0.1011	0.2296	-0.0777	0.4065	1.0000	0.1874	-0.4863	0.2871	-0.2454	0.3552	-0.5761	0.2788	0.7734	0.3725	**DGR-VP1**
**Npun_F4890**	0.0000	0.7616	0.7382	0.5562	0.7399	0.1874	1.0000	0.0713	0.3338	0.1903	0.5653	-0.1639	0.0504	0.4925	0.2729	**DGR-VP2**
**Npun_F4891**	0.7209	0.2538	0.3127	0.3386	0.3002	-0.4863	0.0713	1.0000	0.3225	-0.2638	0.3243	0.4269	-0.4950	-0.2210	0.0359	**DGR-Avd**
**Npun_F4892**	0.0637	0.1663	0.4173	0.1605	0.6518	0.2871	0.3338	0.3225	1.0000	0.1369	0.4499	-0.5021	-0.2648	0.3966	0.7839	**DGR-RT**
**DGR-TR**	-0.4366	0.2213	-0.0526	0.1880	-0.0192	-0.2454	0.1903	-0.2638	0.1369	1.0000	-0.0581	-0.1546	-0.1065	-0.1759	0.2404	**DGR-TR**
Npun_F4893	0.3529	0.3876	0.5285	0.4294	0.6820	0.3552	0.5653	0.3243	0.4499	-0.0581	1.0000	-0.1006	-0.4077	0.3684	0.4785	HTH Regulator
Npun_F4894	0.2061	-0.0107	-0.2514	-0.0774	-0.3750	-0.5761	-0.1639	0.4269	-0.5021	-0.1546	-0.1006	1.0000	-0.0232	-0.6033	-0.4418	Hypothetical protein
Npun_F4895	-0.3143	0.0355	0.0305	0.0242	0.0369	0.2788	0.0504	-0.4950	-0.2648	-0.1065	-0.4077	-0.0232	1.0000	0.4298	-0.3552	Hypothetical protein
Npun_F4896	0.0880	0.3152	0.6137	0.4239	0.7537	0.7734	0.4925	-0.2210	0.3966	-0.1759	0.3684	-0.6033	0.4298	1.0000	0.1685	Helicase
Npun_F4897	-0.2436	-0.1244	0.0696	-0.1876	0.3606	0.3725	0.2729	0.0359	0.7839	0.2404	0.4785	-0.4418	-0.3552	0.1685	1.0000	Restriction endonuclease
Annotation	Hypothetical protein	CHAT domain protein	Hypothetical protein	MoxR ATPase	VWA protein	**DGR-VP1**	**DGR-VP2**	**DGR-Avd**	**DGR-RT**	**DGR-TR**	HTH Regulator	Hypothetical protein	Hypothetical protein	Helicase	Restriction endonuclease	
